# *RNA‐Seq‐Pop*: Exploiting the sequence in RNA sequencing—A Snakemake workflow reveals patterns of insecticide resistance in the malaria vector *Anopheles gambiae*

**DOI:** 10.1111/1755-0998.13759

**Published:** 2023-02-10

**Authors:** Sanjay C. Nagi, Ambrose Oruni, David Weetman, Martin J. Donnelly

**Affiliations:** ^1^ Department of Vector Biology Liverpool School of Tropical Medicine Liverpool UK; ^2^ Uganda Virus Research Institute Entebbe Uganda

**Keywords:** *Anopheles*, insecticide resistance, population genetics, Snakemake, transcriptomics

## Abstract

We provide a reproducible and scalable Snakemake workflow, called *RNA‐Seq‐Pop*, which provides end‐to‐end analysis of RNA sequencing data sets. The workflow allows the user to perform quality control, perform differential expression analyses and call genomic variants. Additional options include the calculation of allele frequencies of variants of interest, summaries of genetic variation and population structure, and genome‐wide selection scans, together with clear visualizations. *RNA‐Seq‐Pop* is applicable to any organism, and we demonstrate the utility of the workflow by investigating pyrethroid resistance in selected strains of the major malaria mosquito, *Anopheles gambiae*. The workflow provides additional modules specifically for *An. gambiae*, including estimating recent ancestry and determining the karyotype of common chromosomal inversions. The Busia laboratory colony used for selections was collected in Busia, Uganda, in November 2018. We performed a comparative analysis of three groups: a parental G24 Busia strain; its deltamethrin‐selected G28 offspring; and the susceptible reference strain Kisumu. Measures of genetic diversity reveal patterns consistent with that of laboratory colonization and selection, with the parental Busia strain exhibiting the highest nucleotide diversity, followed by the selected Busia offspring, and finally, Kisumu. Differential expression and variant analyses reveal that the selected Busia colony exhibits a number of distinct mechanisms of pyrethroid resistance, including the Vgsc‐995S target‐site mutation, upregulation of SAP genes, P450s and a cluster of carboxylesterases. During deltamethrin selections, the 2La chromosomal inversion rose in frequency (from 33% to 86%), supporting a previous link with pyrethroid resistance. *RNA‐Seq‐Pop* is hosted at: github.com/sanjaynagi/rna‐seq‐pop. We anticipate that the workflow will provide a useful tool to facilitate reproducible, transcriptomic studies in *An. gambiae* and other taxa.

## 1 INTRODUCTION

Transcriptomics is central to our understanding of how genetic variation influences phenotype (Stark et al., 2019). In recent years, RNA‐sequencing (RNA‐Seq) has replaced microarray technologies for whole‐transcriptome profiling, providing a relatively unbiased view of transcript expression (Zhao et al., 2014) with associated higher sensitivity and greater dynamic range (Lowe et al., 2017). The utility of RNA‐Seq is exemplified by the vast amounts of data accruing (Van den Berge et al., 2019), and in the many discoveries it has revealed—such as the extent of alternative splicing, and the biology of noncoding RNAs (Stark et al., 2019; Wang et al., 2010; Wang & Burge, 2008).

In recent years, various computational workflows have been developed to analyse RNA‐Seq data in a reproducible manner (Lataretu & Hölzer, 2020; Zhang & Jonassen, 2020), but these workflows are designed with the primary aim of differential expression analysis (DEA) and leave a large amount of untapped sequence‐based information. A study previously detected population genomic signals in RNA‐Seq data, although this specific application remains rare (Thorstensen et al., 2020). In our own area of research, vector genomics, a scan of the literature revealed 33 RNA‐Seq studies (Table S1), of which only five interrogated the sequence data (Bonizzoni et al., 2015; David et al., 2014; Faucon et al., 2017; Kang et al., 2021; Messenger et al., 2021). A barrier to exploiting the full range of information contained within RNA‐Seq data sets has been the absence of comprehensive, user‐friendly pipelines which permit easily reproducible analysis (Grüning et al., 2018) and enable comparisons across studies.

In this study, using the workflow management system Snakemake (Mölder et al., 2021), we present a reproducible computational workflow, *RNA‐Seq‐Pop*, for the analysis of short‐read RNA‐Seq data sets of any organism. The workflow is applicable to single or paired‐end RNA‐Seq data, such as those produced on Illumina or MGI (DNB‐Seq) instruments. However, we also present modules specifically of interest in the analysis of the major malaria mosquito, *Anopheles gambiae s.l*., and demonstrate their use in a study of pyrethroid‐resistance in a strain of *An. gambiae* from Busia, Uganda.

Pyrethroids are the most widely used class of insecticide in malaria control, and over the past two decades, resistance in malaria vectors has spread throughout sub‐Saharan Africa, posing a threat to vector control efforts (Ranson, 2017). In this period, the incrimination of genes involved in insecticide‐resistant phenotypes of *An. gambiae* has been based primarily on transcriptomic studies. For many years, these were performed using microarrays, synthesis of which has highlighted the repeatable overexpression of a handful of genes involved in detoxification, confirming well‐established cytochrome P450s as candidates, whilst also implicating more diverse genes such as ABC transporters and sensory appendage proteins (Müller et al., 2008, Ingham et al., 2018). Yet to date, relatively few diagnostic markers have been identified, and important genes have been missed by standard transcriptomic analyses (Njoroge et al., 2022). These shortcomings illustrate the need for a more comprehensive approach to marker discovery. While whole‐genome sequencing is providing valuable information on known and novel resistance variants (Clarkson et al., 2021; The Anopheles gambiae 1000 Genomes Consortium, 2020), exploiting the sequence data within RNA‐Seq can help bridge the step from transcriptomics to genomics.

In Uganda, pyrethroid resistance has escalated in recent years (Lynd et al., 2019; Tchouakui et al., 2021). As well as the *Vgsc*‐995S mutation, which has repeatedly been associated with pyrethroid resistance, recent genomic studies from this region have shown that a triple‐mutant haplotype, linking a transposable element, a gene duplication (*Cyp6aa1*) and a nonsynonymous mutation *Cyp6p4*‐I236M, is an important marker of pyrethroid resistance (Njoroge et al., 2022). A single nucleotide polymorphism (SNP)‐array based genome‐wide association study (GWAS) also demonstrated the *Cyp4J5*‐L43F mutation to be a useful marker for insecticide resistance, whilst also implicating the 2La inversion karyotype as a potential marker (Weetman et al., 2018). We use *RNA‐Seq‐Pop* to uncover patterns of insecticide resistance in Ugandan *An. gambiae*, monitoring these resistance‐associated mutations, whilst performing differential expression analyses, summarizing genetic variation and ancestry, and karyotyping chromosomal inversions.

## 2 MATERIALS AND METHODS

### 2.1 *RNA‐Seq‐Pop* implementation

**FIGURE 1:**
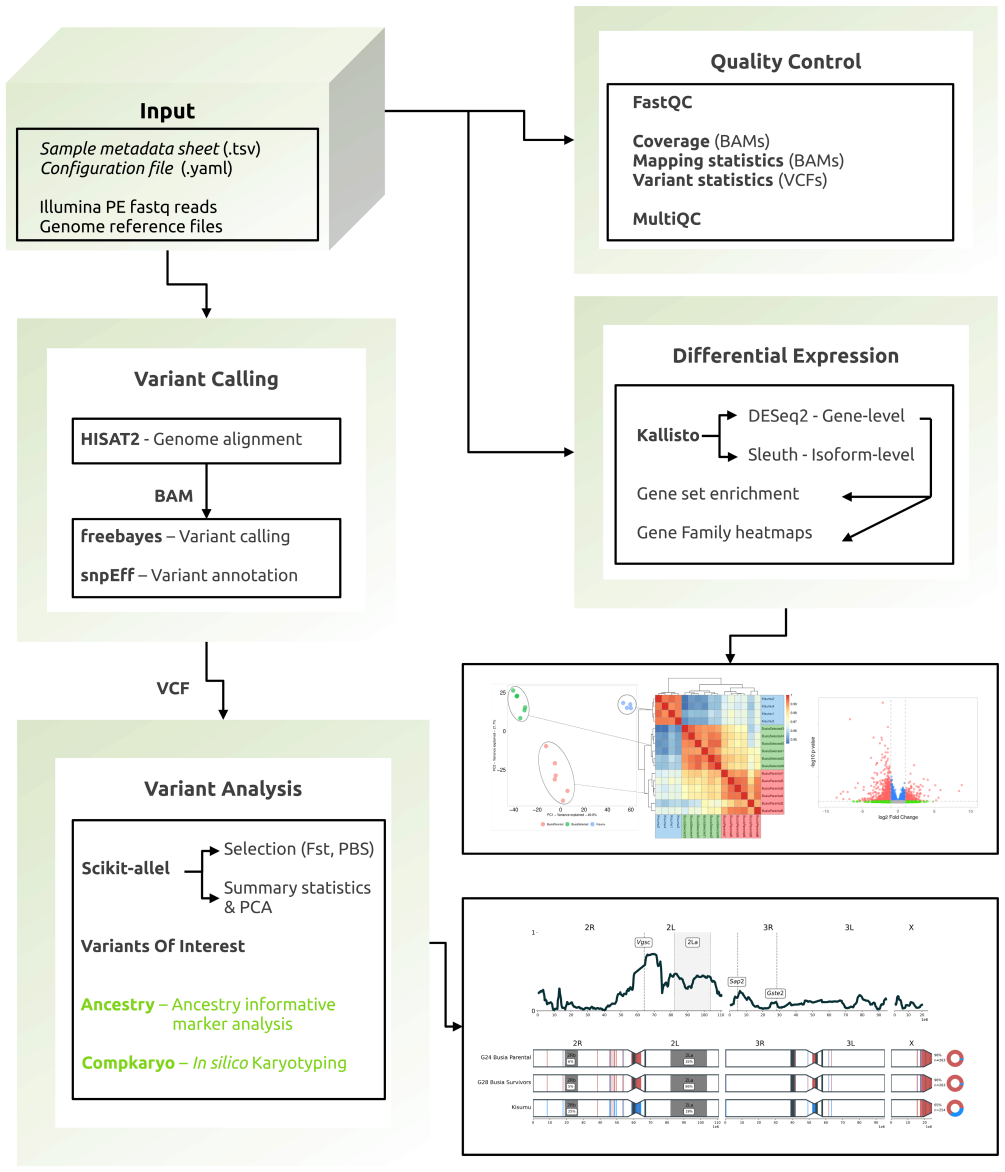
The *RNA‐Seq‐Pop* workflow and example outputs. The workflow has been designed for ease of use, requiring only a configuration file to set up workflow choices and a sample sheet to provide sample metadata. Modules highlighted in green are specific to *Anopheles gambiae s.l*.

We designed the *RNA‐Seq‐Pop* workflow according to Snakemake best practices (Köster, 2022). *RNA‐Seq‐Pop* is constructed with a single configuration file in human‐readable yaml format (the config file), alongside a simple tab‐separated text file containing sample metadata (the sample sheet). The overall *RNA‐Seq‐Pop* workflow is shown in Figure 1.

Dependencies are internally managed by the package manager conda; to instal all required software, specify the ‐‐use‐conda directive at the command line, and conda will automatically create isolated software environments in which to run. As of version 1.0.4, *RNA‐Seq‐Pop* modules are written in Python (81.2% of the codebase) and R (18.8%), and internally, the workflow utilizes a library (RNASeqPopTools) which provides the infrastructure to the Python codebase, to ensure readability. We provide documentation to guide users on how to set up and run *RNA‐Seq‐Pop*: https://sanjaynagi.github.io/rna‐seq‐pop.

#### 2.1.1 Quality control

The workflow begins by checking concordance between the user‐provided sample metadata, configuration file, and reference and fastq files. Quality control metrics of fastq files are calculated with fastqc (Andrews, 2010), and logs and statistics from eight tools in the workflow are integrated into a report with multiqc (Ewels et al., 2016). Raw fastq reads may be optionally trimmed with cutadapt (Martin, 2011), with the option of specifiying custom adaptor sequences.

#### 2.1.2 Differential expression

Trimmed reads are aligned to the reference transcriptome with kallisto (Bray et al., 2015) and differential expression performed at the gene level with deseq2 (Love et al., 2014) and at the isoform level with sleuth (Pimentel et al., 2017). The gene‐level counts are normalized to account for sequencing depth, and principal components analysis (PCA) and Pearson's correlation performed among all samples, and on subsets of the user‐selected treatment groups used in differential expression analysis. Plots of these analyses are useful for exploratory data visualization, providing an additional quality control step to ensure expected relationships between samples. *RNA‐Seq‐Pop* combines differential expression results from multiple pairwise comparisons into an excel spreadsheet for the user, as well as generating individual .csv files, volcano plots, and identifying the number of differentially expressed genes at various false discovery rate (FDR)‐adjusted *p*‐value thresholds. The user may create venn diagrams for multiple comparisons and generate heatmaps if a list of genes is provided. We use the hypergeometric test for Gene Ontology (GO) term enrichment analysis, on genes that are significantly over‐expressed based on FDR‐adjusted *p*‐values and, and optionally, the top five percentile of *F*_ST_ values.

#### 2.1.3 Variant calling

Reads are aligned to the reference genome with hisat2 (Kim et al., 2019) and read duplicates marked with samblaster (Faust & Hall, 2014) producing binary alignment files (BAM), which are sorted by genomic coordinate and indexed with samtools version 1.19 (Danecek et al., 2021). SNPs are then called with the Bayesian haplotype‐based caller freebayes version 1.3.2 (Garrison & Marth, 2012). SNPs are called jointly on all samples, with different treatment groups called as separate populations, at the ploidy level provided by the user in the configuration file. The workflow internally parallelizes freebayes by splitting the genome into small regions, greatly reducing overall computation time. The separated genomic regions are then concatenated with bcftools version 1.19 (Danecek et al., 2021) and the final VCF piped through vcfuniq (Garrison et al., 2021), to filter out any duplicate calls that may occur at the genomic intervals between chunks. Called variants are then annotated using snpeff version 5.0 (Cingolani et al., 2012).

#### 2.1.4 Variant analysis and selection

*RNA‐Seq‐Pop* can then perform analyses on the variants called by freebayes. We apply filters to the data, including restricting to SNPs (excluding indel calls) and applying missingness and quality filters. We recommend using a quality score of 30 and a missingness proportion of 1, meaning a variant call (reference or alternate allele) must be present in each sample, that is there are no missing allele calls. For each pairwise comparison specified in the config file, the workflow can perform a windowed Hudson's *F*_ST_ scan (Bhatia et al., 2013; Hudson et al., 1992) along each chromosomal arm, outputting windowed *F*_ST_ estimates and genome‐wide plots. Population branch statistic (PBS) scans may also be performed, conditional on the presence of three suitable populations for the phenotype(s) of interest (Yi et al., 2010). It is also possible to run Hudson's *F*_ST_ and PBS scans, taking the average for each protein‐coding gene, rather than in windows. All population genetic statistics are calculated in scikit‐allel version 1.2.1. (Miles & Harding, 2017). We also provide a script (geneScan.py) to interrogate the VCF files, reporting missense variants from any gene of the user's choice. A tab‐separated file of variants of interest can be provided, from which the workflow will produce allele frequency heatmaps for each biological replicate and averaged across treatment groups. We define the expressed allele balance as the allele frequency at a genomic location in the aligned read data—for this analysis, *RNA‐Seq‐Pop* does not use variants called by freebayes, but instead calculates the proportion of each allele directly in bam files to ease intepretation. An example variant of interest file for *Anopheles gambiae* is provided in the *RNA‐Seq‐Pop* GitHub repository.

All analyses described thus far can be conducted across all eukaryotes of any ploidy, requiring only a reference genome (.fa), transcriptome (.fa) and genome annotation files (.gff3).

#### 2.1.5 *Anopheles gambiae s.l.* specific analyses

For *An. gambiae s.l*. data sets we have exploited the *Anopheles gambiae* 1000 genomes resource (Miles et al., 2017; The Anopheles gambiae 1000 Genomes Consortium, 2020), to incorporate H12 and iHS (Garud et al., 2015) genomic selective sweep analysis. The workflow outputs the differentially expressed gene's genomic location, the specific sweep signals that are present in the Ag1000g resource at that genomic location, and whether the region is a known insecticide resistance‐associated locus. We caution that this kind of analysis is exploratory: many genes will be contained within selective sweeps, and may not have a causal link to phenotypic variation.

#### 2.1.6 Population structure, ancestry and karyotyping

To investigate population structure, we apply SNP quality and missingness filters to the SNP data, which can be configured by the user. Multiple measures of population genetic diversity are estimated for each sample, such as nucleotide diversity (*π*), Watterson's *θ* (Watterson, 1975) and inbreeding coefficients. We then prune SNPs in high linkage by excluding variants above an *R* ^2^ threshold of .01 in sliding windows of 500 SNPs with a step size of 250 SNPs, and perform a PCA on the remaining SNPs. If the analysed species is *An. gambiae*, *An. coluzzii* or *An. arabiensis*, the pipeline can implement an analysis of putative ancestry informative markers (AIMs). The AIMs were derived from two different data sets. The *An. gambiae*/*An. coluzzii* AIMs derive from the 16 genomes project (Neafsey et al., 2015) and in West Africa may distinguish between individuals with *An. gambiae* or *An. coluzzii* ancestry. The *An. gambcolu*/*An. arabiensis* AIMs are derived from phase 3 of the *Anopheles gambiae* 1000 genomes project, and distinguish between individuals with either *An. gambiae* or *An. coluzzi* ancestry from *An. arabiensis*. The relative proportion of ancestry is reported and visualized for the whole genome by chromosome. We modified the program compkaryo (Love et al., 2019) to enable the identification of common inversions on chromosome 2 in pooled samples.

### 2.2 Busia RNA‐Seq

#### 2.2.1 Mosquito lines

As a case study to the workflow, we sequenced a pyrethroid‐resistant colony of *An. gambiae s.s*. from Busia, Uganda, alongside the standard multi‐insecticide‐susceptible reference strain, Kisumu. After 24 generations in the colony, we stored RNA from unexposed Busia individuals (G24 Busia parental). Unexposed, age‐matched Kisumu females were used as controls. We then selected the remaining Busia G24 colony using 0.05% deltamethrin papers in WHO tube assays for four generations (full details of the selection regime can be found in the Text S2). We exposed females from the selected generation (G28) for 1 h to 0.05% deltamethrin WHO papers using standard protocols, left for 24 h after exposure, and survivors were stored at −80°C prior to RNA extraction (G28 Busia selected survivors). Selections were perfomed following mating.

#### 2.2.2 Library preparation

We extracted RNA from pools of five, 4‐day‐old female mosquitoes using a Picopure RNA isolation kit (Arcturus, Applied Biosystems). We performed six replicates for each Busia‐derived treatment group, and four for Kisumu. Library quality and quantity were determined on a Tapestation 2200 (Agilent) using high‐sensitivity RNA screentape. Paired‐end 150‐bp RNA‐Seq libraries were prepared and sequenced by Novogene (https://en.novogene.com/), on an Illumina NovaSeq 6000 system.

## 3 RESULTS

### 3.1 Busia resistance phenotyping

The parental G24 *Anopheles gambiae* Busia strain had lost much of its pyrethroid resistance during the time in culture and exhibited susceptibility to deltamethrin (100% mortality, 96.3–100 95% confidence intervals [CIs]) and low‐prevalence resistance to permethrin (92.6% mortality, 85.6–96.4 95% CIs). Four generations of deltamethrin selections demonstrated this loss to be readily reversible and resulted in a G28 selected Busia strain that showed increased resistance to both deltamethrin (69.7% mortality, 63.2–75.6 95% CIs) and permethrin (21.7% mortality, 14.9–30.5 95% CIs) when exposed for 1 h in WHO tube assays. Further colony information can be found in Table S2. We compared the G24 Busia parental strain with the G28 Busia selected strain, and both Busia strains to the pyrethroid‐susceptible reference strain, Kisumu.

### 3.2 RNA‐Seq

As an illustrative example of the modules and output of the *RNA‐Seq‐Pop* workflow, we will describe the analysis of the Busia RNA‐Seq data set.

#### 3.2.1 Quality control

**FIGURE 2:**
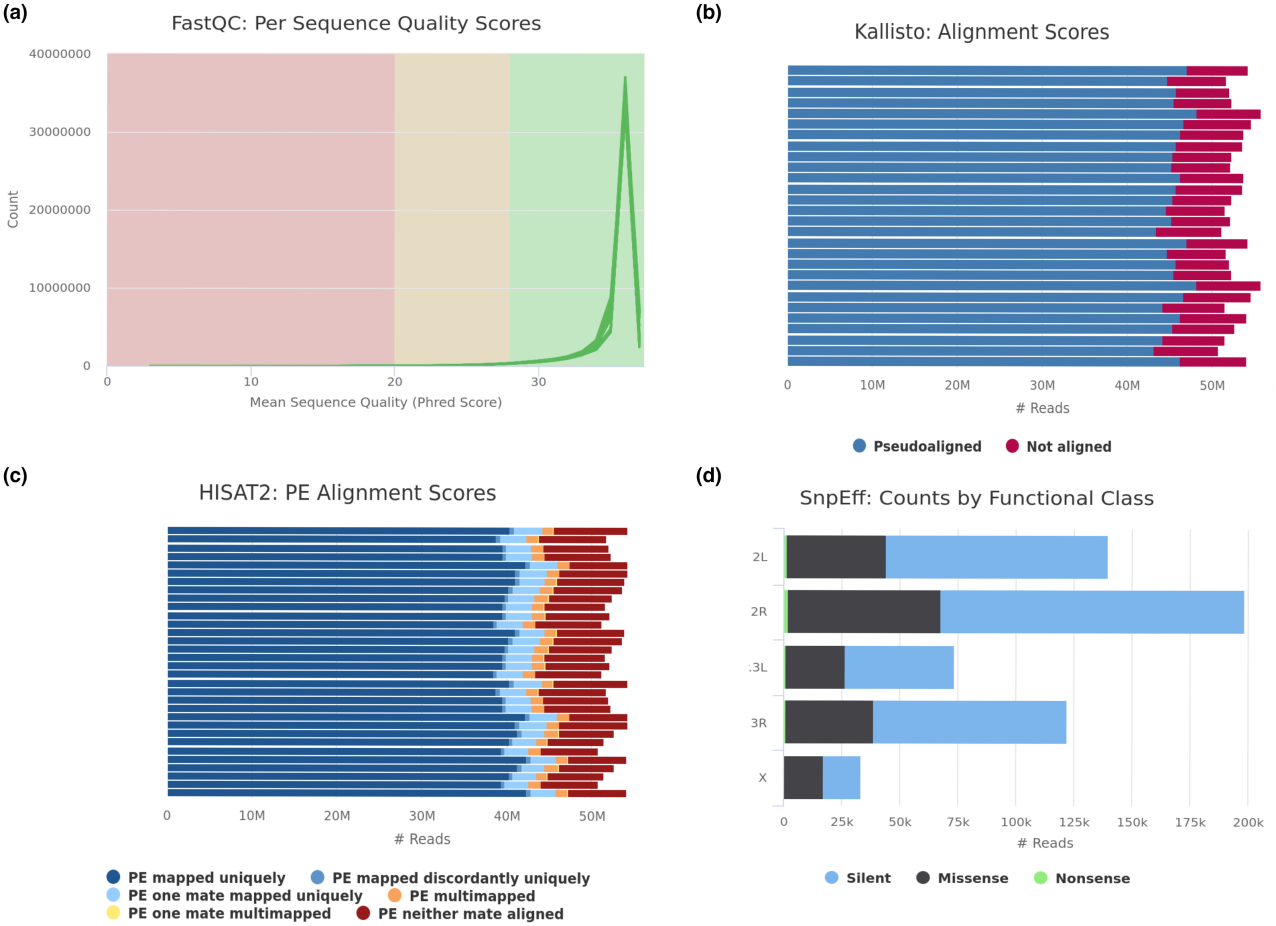
multiqc captures quality control statistics from across the *RNA‐Seq‐Pop* workflow. (a) Per‐base sequence content as calculated by fastqc. (b) Total reads and number of successfully aligned reads to the reference transcriptome by kallisto. (c) The number of reads that were successfully mapped to the reference genome with hisat2. (d) The proportion of missense, synonymous and nonsense SNPs reported by snpeff.

We used *RNA‐Seq‐Pop* to import FASTQ data files into fastqc (Andrews, 2010) to determine levels of adaptor content, quality scores, sequence duplication levels and GC content in the raw read data. After genome alignment, we applied rseqqc and samtools to collect mapping statistics from the resulting BAM files. We then integrated multiqc into the workflow, which collates statistics and results from eight tools to generate a convenient, interactive (.html) quality control report. Figure 2 shows reports generated by multiqc on the Busia *An. gambiae* data set.

We removed adapter sequences and aligned paired‐end reads to the *An. gambiae* PEST reference transcriptome (AgamP4.12) (Figure 2b). In total, 844.25 million reads were processed in total, with 727.84 million successfully aligned, giving an overall alignment rate of 85.58% (±0.206% standard error) across 16 replicates in total. The breakdown of reads counted per sample can be found in Figure S3.

**FIGURE 3:**
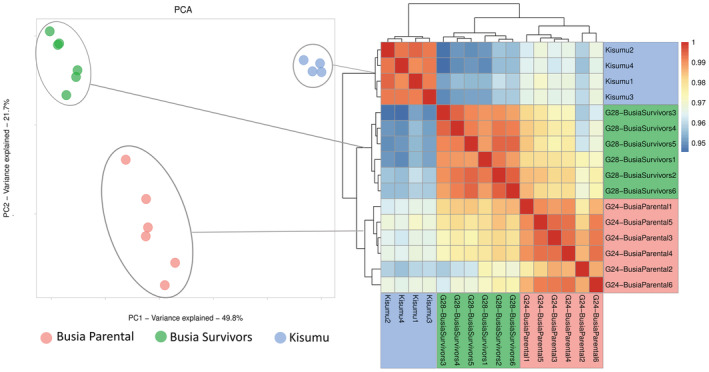
Exploratory sample clustering. (a) Principal components analysis of the normalized read count data, showing clear separation between conditions. (b) A sample‐to‐sample Pearson's correlation heatmap of normalized read counts assigned to each biological replicate; dendrograms show hierarchical clustering applied directly to Pearson's correlations.

As a further quality control step, and to uncover the overarching relationships of gene expression between samples, *RNA‐Seq‐Pop* performs a PCA (Figure 3a), and a sample‐to‐sample correlation heatmap (Figure 3b) on the deseq2‐normalized read count data. In both analyses, biological replicates of each treatment group clustered together, supporting robust replication in these samples.

#### 3.2.2 Differential expression

We compared the G24 parental Busia strain to the G28 Busia survivors, and also to the laboratory‐susceptible Kisumu, which provides a cross‐reference with earlier studies, as well as an extra level of filtering to identify candidate genes. Using an adjusted *p*‐value threshold of .05, our deseq2 differential expression analysis (Wald test) identified 5416 differentially expressed genes between Kisumu and the parental Busia line and 5657 between the parental Busia and the G28 selected Busia survivors. The full table of differentially expressed genes in all comparisons can be found in Data S1, and volcano plots in Figure S4a–c.

After four generations of selections plus insecticide exposure, a number of genes belonging to candidate detoxification families were significantly differentially expressed between the G24 Busia parental and G28 Busia selected strains (e.g., 51 cytochrome P450s, 23 carboxylesterases and 20 ABC transporters). All three sensory appendage protein (*Sap*) genes in the *An. gambiae* genome were significantly overexpressed in the G28 Busia selected survivors compared to the parental Busia line. *Sap2* showed 10.7‐fold overexpression (6.5–17.5 95% CIs), while *Sap1* exhibited 1.8‐fold (1.36–2.44 95% CIs) and *Sap3* 2.0‐fold (1.58–2.51 95% CIs) overexpression.

**FIGURE 4:**
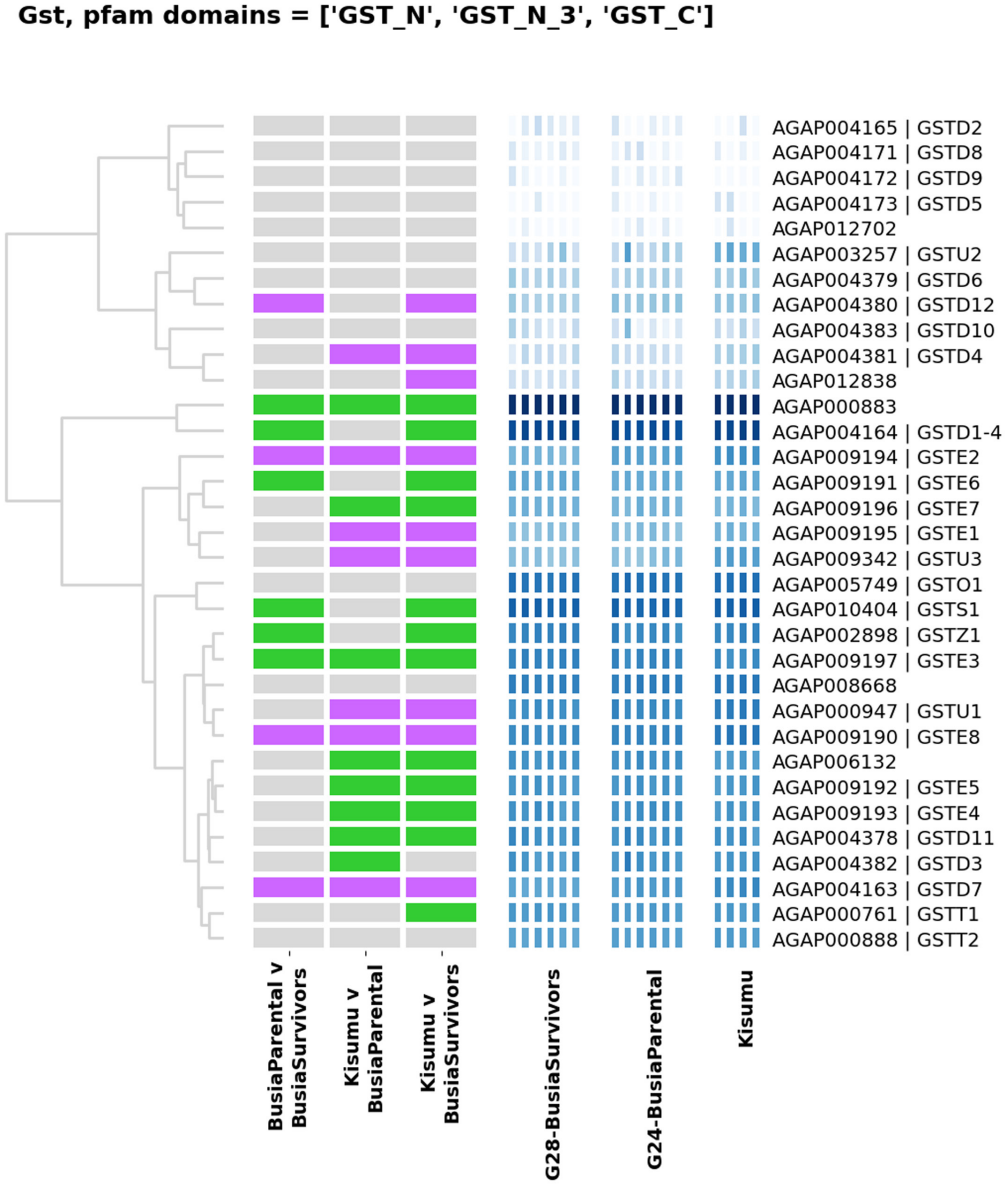
Summary of gene expression in gene families of interest. Using PFAM domains, we extract genes from specific gene families, and summarize fold‐change (left) and normalized read count data (right). Genes are ordered by hierarchical clustering of the normalized read count data, and clustering results are displayed by the dendrogram (far left). In the fold‐change plot (left), green = overexpression in the second group, and purple = underexpression.

We also provide a module which summarizes gene expression in specific gene families if provided with a table mapping genes to pfam domains. We provide an example mapping file. Figure 4 shows a summary of expression data in the glutathione‐S‐transferase (GST) gene family, known to be associated with insecticide resistance. The normalized read counts for each gene (blue squares) are clustered and ordered with hierarchical clustering. We then plot the clustering dendrogram alongside a summary of differential expression in each comparison, and the normalized read counts for each biological replicate. Further plots for other gene families are provided in Data S3. The user may specify their own pfam domains of interest. The default settings apply the analysis to cytochrome P450s, GSTs, carboxylesterases, ABC‐transporters, odorant binding proteins, olfactory receptors, ionotropic receptors, gustatory receptors and cuticle‐related genes. In addition to this module, the user may also supply a list of transcripts, and *RNA‐Seq‐Pop* will produce a heatmap on the normalized read count data (Figure S5).

To investigate the similarity of differential expression comparisons, *RNA‐Seq‐Pop* provides a venn diagram module which displays the number of shared up‐ or downregulated genes between multiple comparisons (Figure S6). Using a separate option within *RNA‐Seq‐Pop* that looks for genes expressed in the same direction across multiple comparisons, we identified a cluster of carboxylesterases which were overexpressed in G24 Busia parental vs. Kisumu and in G28 Busia selected survivors vs G24 Busia parental. In the latter comparison, *Coebe2c* showed a fold change of 1.69 (1.3–2.1 95% CIs), *Coebe3c* 3.05 (1.6–5.9 95% CIs) and *Coebe4c* 1.61 (1.2–2.2 95% CIs). We examined whether any selective sweeps were observed around these loci in the Ag1000g phase 1 data set and identified one in a population of *An. gambiae* from Gabon, though not in Uganda.

*RNA‐Seq‐Pop* also performs differential expression at the isoform level with sleuth. As an example, we examined isoform‐level variation at the voltage‐gated sodium channel (*Vgsc*), the target of pyrethroid insecticides. As *Vgsc* contains 13 annotated transcripts and 39 exons, there is a large potential for alternative splicing, which could be an important but as of yet overlooked mechanism of target‐site resistance. Between the Busia G24 parental strain and the Busia G28 survivors, we found no significant difference in expression of any *Vgsc* transcript. However, when comparing the susceptible Kisumu strain to the G24 Busia parental strain, five *Vgsc* transcripts were differentially expressed—AGAP004707‐RA (adjusted *p* = .0059), AGAP004707‐RD (adjusted *p* = .0096), AGAP004707‐RI (adjusted *p* = .0095), AGAP004707‐RL (adjusted *p* = 1.4e^−12^) and AGAP004707‐RM (adjusted *p* = 4.36e^−07^). Given the minimal phenotypic difference between these two strains, it is not clear whether these differences are related to pyrethroid resistance or if this variation is natural between strains.

#### 3.2.3 Variant calling

**FIGURE 5:**
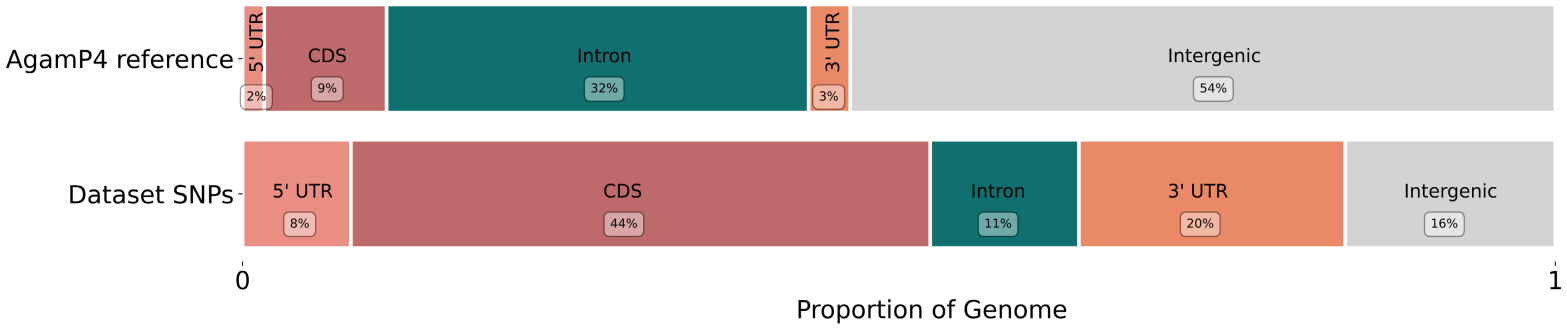
SNPs from RNA‐Seq are enriched in transcribed regions. Illustration of the proportion of SNPs found within each genomic feature in the AgamP4 reference genome (upper panel) and in the combined Busia and Kisumu *Anopheles gambiae* RNA‐Seq data set (lower panel).

We enabled *RNA‐Seq‐Pop* to call genomic variants with freebayes and output data in VCF format. Across all chromosomes, and after filtering, *RNA‐Seq‐Pop* called 734,269 variants. Figure 5 provides a visual representation of genome composition in the *An. gambiae* PEST reference genome, and the proportion of SNPs covered by each genomic feature in our genotype calls. The *An. gambiae* genome consists of 54% intergenic and 46% genic sequence (of which 14% are exonic and 32% intronic). Given the nature of RNA‐Seq, we expected to primarily find SNPs in coding regions of the genome, which are expressed. Indeed, of these 734,269 variants, we find 73% residing within exons, 11% in introns and 16% in intergenic regions. The finding of 16% of SNPs in intergenic regions is likely to be explained by expression of noncoding RNAs, and the misannotation of transcripts—particularly 5′ and 3′ untranslated regions (UTRs). The workflow automatically annotates the called variants with snpEff—across all exons, 16.4% of variants were annotated as nonsynonymous and 58.1% as synonymous.

There was a positive correlation between read counts per gene and the number of called SNPs per gene when controlling for gene size (GLM: coef = .135, *p* = 2.2e^−36^, Table S5). A PCA based upon read count data was not qualitatively different from the PCA on expression data (Figure 3 and Figure S8).

#### 3.2.4 Genetic diversity

Table 1 shows genome‐wide nucleotide diversity (*π*) and Watterson's *θ*, averaged across 20‐kb nonoverlapping windows. To standardize sample size we down‐sampled both Busia strains from six to four replicates. Both measures of genetic diversity were significantly lower in the Kisumu strain compared to the two Busia strains, as would be expected after a long history of laboratory colonization. The G28 selected Busia survivors also showed a reduction in genetic diversity compared to the unexposed G24 parental Busia colony.

**TABLE 1:** Genetic diversity.

	*π* (95% CIs)	Θ (95% CIs)
G24 Busia parental	1.04 × 10^−3^ (1.02 × 10^−3^–1.07 × 10^−3^)	7.4 × 10^−4^ (7.23 × 10^−4^–7.57 × 10^−4^)
G28 Busia survivors	7.07 × 10^−4^ (6.87 × 10^−4^–7.27 × 10^−4^)	5.51 × 10^−4^ (5.37 × 10^−4^–5.65 × 10^−4^)
Kisumu	6.18 × 10^−4^ (6.0 × 10^−4^–6.35 × 10^−4^)	4.06 × 10^−4^ (3.95 × 10^−4^–4.18 × 10^−4^)

*Note*: Average measures of genetic diversity, calculated in 20‐kb overlapping windows, across chromosomal arms: *π*, nucleotide diversity; Θ, Watterson's theta.

#### 3.2.5 Known insecticide resistance variants of interest

**FIGURE 6:**
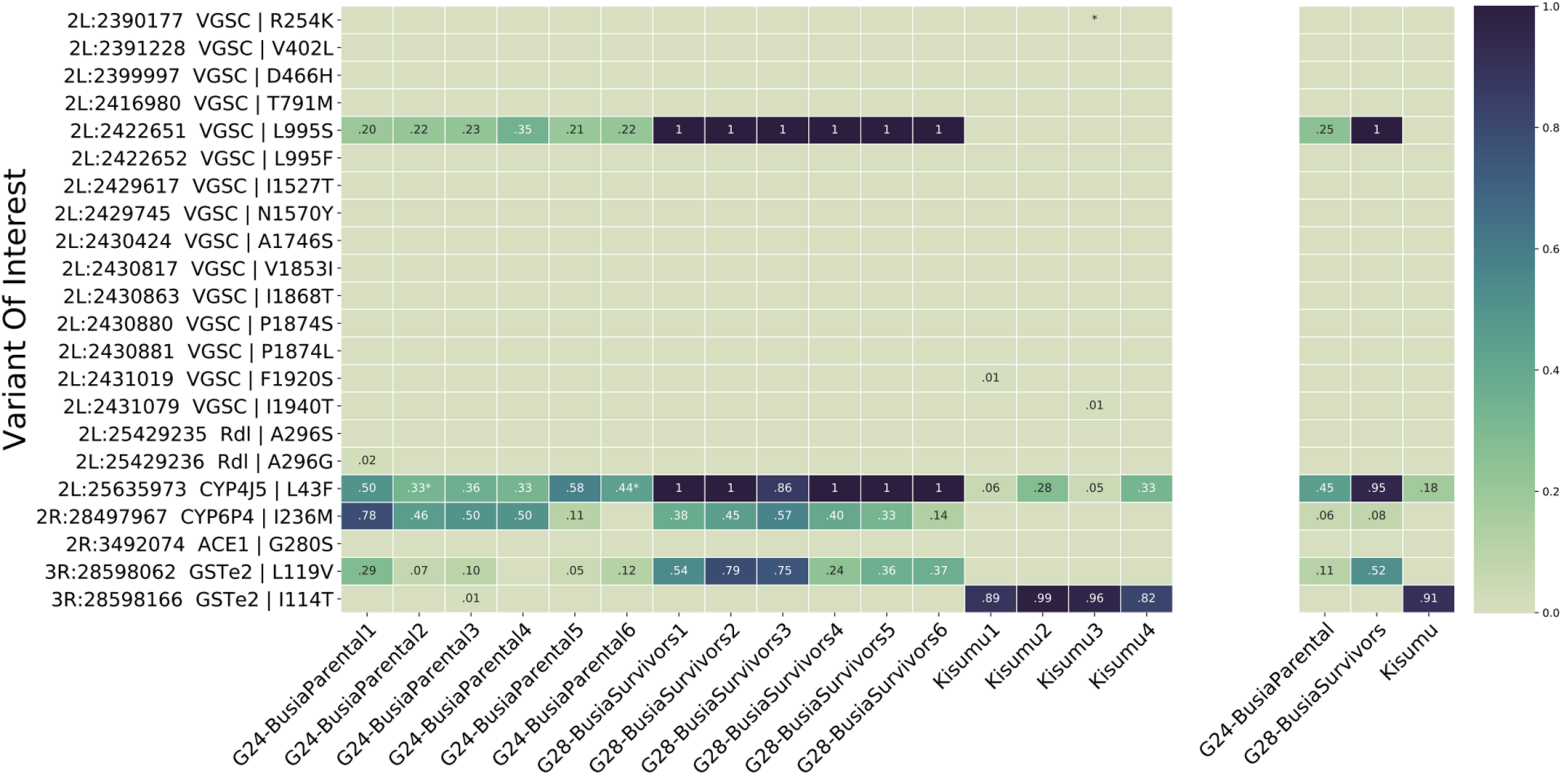
Variants of interest. A heatmap showing allele frequencies of variants of interest found in read data in (a) each sample and (b) overall average allele frequency across strains. Blank cells indicate that the mutant allele was not detected despite reads across that genomic position.

If provided with a list of user‐defined variants of interest, *RNA‐Seq‐Pop* will generate reports and plots of allele balance (the allele frequency found in the read alignments). For our variants of interest, we curated a selection of SNPs which have been associated with insecticide resistance in previous studies. Figure 6 shows allele frequencies of variants of interest across all samples. We show that over the four generations of selections and after insecticide exposure, the frequency of the *Vgsc*‐995S *kdr* allele increased from 25% (95% CIs: 21.5%–29.8%) in G24 to 100% in the G28 Busia survivors. In agreement with recent work from the Ag1000g project, we found no known secondary *kdr* mutations alongside the *Vgsc*‐995S allele (Clarkson et al., 2021).

In addition, the *Cyp4j5*‐L43F mutation, previously associated with insecticide resistance in Uganda, showed a large increase in frequency after the selection regime and exposure, increasing from an average frequency of 45% (95% CIs: 32%–54%) in the G24 Busia to 95% (95% CIs: 93%–100%) in the G28 Busia survivors. The *Gste2*‐I114T mutation, associated with DDT resistance, was absent in both Busia strains, but surprisingly it was present at high frequency (92%) in the pyrethroid‐susceptible Kisumu reference strain. Another mutation, *Gste2*‐L119V, increased in frequency from 11% (95% CIs: 9%–13%) to 52% (95% CIs: 47%–58%). The *Cyp6p4*‐I236M mutation, linked to a swept haplotype in Uganda, was present in Busia samples, but there was no significant difference in frequency between the parental (39%, 95% CIs: 29%–53%) and selected survivors (38%, 95% CIs: 26%–52%). In agreement with these differences in frequency of known insecticide‐resistance variants, we find *F*_ST_ values in both the *Vgsc* and *Cyp4J5* genes in the top 5th percentile between the G24 parental Busia strain and the G28 selected Busia survivors, but not in *Cyp6P4* (89th percentile).

The *Ace‐1*‐G280S mutation was absent from all samples. A single allele of the *rdl*‐A296G mutation was detected in the parental Busia strain. Complete allele balance data for all variants of interest can be found in Data S2. We looked within the primary candidate gene from differential expression analysis, *Sap2*, for allele frequency changes, but no nonsynonymous variants were present in the data.

#### 3.2.6 Selection

**FIGURE 7:**
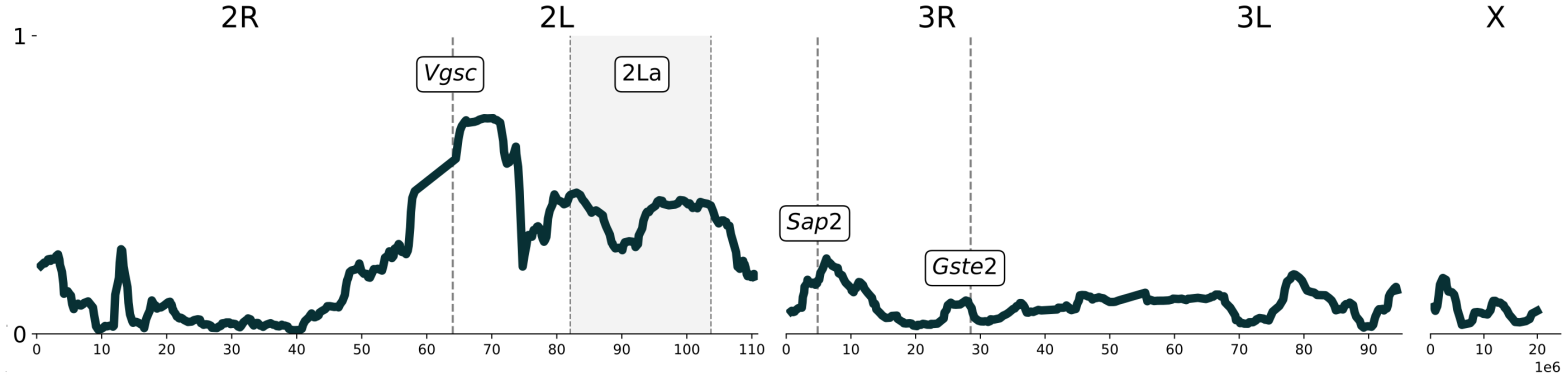
Selection. Hudson's *F*_ST_ calculated in windows across the genome, comparing the G24 Busia parental strain to the G28 Busia survivors. High genetic differentiation can be observed on the 2L chromosomal arm.

The workflow permits calculation of *F*_ST_ and PBS both in windows as genome‐wide selection scans (GWSS) and within each gene. In the context of insecticide resistance, finding regions of high genetic differentiation between susceptible and resistant mosquito populations can allow us to identify loci or variants that contribute to the phenotype. We found high overall levels of *F*_ST_ between the G24 parental Busia and the G28 selected Busia survivors, but *F*_ST_ on chromosomal arm 2L was especially elevated as compared to the other arms (Figure 7), with large *F*_ST_ signals around the *Vgsc* and 2La inversion. In other data sets from F_1_ *An. gambiae* (examples in Figure S13), the genome‐wide selection scans are able to capture signals at sites of known selective sweeps.

#### 3.2.7 Chromosomal inversions

We estimated the karyotype of the samples with compkaryo for the 2La and 2Rb chromosomal inversions, by extracting karyotype‐tagging SNPs. Karyotyping‐tagging SNPs are alleles located within the inversion breakpoints, which show fixed (or almost fixed) differences between the inverted and standard karyotypes. We focus on these two inversions because both contain a large number of tagging SNPs, providing confidence in the overall calls. Figure 7 shows a diagram of the *An. gambiae* genome, with the location and average karyotype frequency per group. The 2La inversion was present at a frequency of 86% in the G28 Busia survivors, compared to 33% in the G24 Busia parental strain (Mann–Whitney U, adjusted *p* = .014), where 0% means no 2La alleles across all tag SNP loci, and 100% means all 2La alleles across all tag SNP loci. The frequency of the 2Rb inversion was also significantly different between Kisumu and both Busia colonies (Mann–Whitney U, adjusted *p* < .05). Figure S12 shows the per‐replicate karyotype frequency.

#### 3.2.8 Ancestry

**FIGURE 8:**
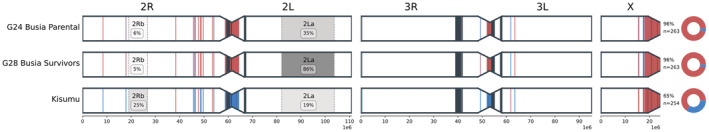
Ancestry and karyotyping. (Left) A diagram of the mosquito chromosomal arms, including heterochromatin regions (black). Ancestry‐informative markers that are indicative of either *Anopheles gambiae* (red) or *An. coluzzii* (blue) are displayed as vertical lines. The major inversions 2La and 2Rb are displayed, along with their respective average frequency among treatment groups, as called by the program compkaryo. (Right) A doughnut chart of the proportion of ancestry‐informative markers that are indicative of either *An. gambiae* (red) or *An. coluzzii* (blue) ancestry for each sample. The overall proportion of gambiae alleles (%) and the number of called AIMs (*n*) per group is labelled.

Ancestry informative markers are SNPs which show fixed (or almost fixed) differences between species. *RNA‐Seq‐Pop* can utilize sets of AIMs to investigate the proportion of ancestry for each chromosome assigned to either *An. gambiae, An. coluzzii* or *An. arabiensis*. Figure 8 shows the position of called AIM alleles that map to either *An. gambiae* or *An. coluzzii* across the genome. This shows that the Busia samples were primarily of *An. gambiae s.s*. ancestry across all chromosomes, in concordance with the X chromosome‐based SINE species ID assay (Santolamazza et al., 2008). However, the pattern was markedly different for the susceptible reference strain, Kisumu, which showed a large degree of putative *An. coluzzii* ancestry on the autosomes (Table S11).

## 4 DISCUSSION

### 4.1 *RNA‐Seq‐Pop* implementation

*RNA‐Seq‐Pop* encompasses a complete workflow for RNA‐Seq analysis, from quality control and read trimming, to transcript quantification and DEA. However, as well as conducting traditional DEAs at both the gene and isoform level, *RNA‐Seq‐Pop* exploits useful, but often ignored sequence data.

*RNA‐Seq‐Pop* is designed for ease of use, requiring only a *sample metadata sheet* and a yaml format *configuration* file. A single command in the terminal will automatically instal all dependencies and run the workflow, which is scaled by Snakemake to run on a personal computer, cluster or cloud environment. The workflow is applicable to single or paired‐end RNA‐Seq data from any organism, allowing for variation in ploidy, including haploid, diploid or pooled samples. We have written *RNA‐Seq‐Pop* in accordance with Snakemake best practices (Köster, 2022), and hope that it is an intuitive program, readily configured by the user to allow reproducible transcriptomic analyses. We present documentation with guidance on how to set up and run the workflow. To increase accessibility, *RNA‐Seq‐Pop* is written in python and R, the two most popular programming languages in the life sciences.

Decreasing sequencing costs have facilitated the proliferation of genomic surveillance in infectious disease research (Neafsey et al., 2021). The specific modules within *RNA‐Seq‐Pop*, which are readily adapted to other organisms, allow us to investigate novel variants that may be involved in our phenotype of interest (insecticide resistance), while simultaneously producing data on known resistance variants which can provide actionable information for malaria control programme personnel. For *Anopheles gambiae s.l*., we provide a versioned variants of interest file in the GitHub repository, which we will update with additional resistance or disease transmission‐related variants. As well as highlighting known variants, *RNA‐Seq‐Pop* can also perform genome‐wide selections scans, using *F*_ST_ (Bhatia et al., 2013) and the PBS (Yi et al., 2010), highlighting known and novel regions of the genome that may be involved in the phenotype of interest.

For the major malaria vector, *An. gambiae s.l*., *RNA‐Seq‐Pop* can determine the karyotype frequency of chromosomal inversions, utilizing the program compkaryo (Love et al., 2019). *An. gambiae s.l*. has been shown to harbour a number of segregating chromosomal inversions, which have been associated with environmental heterogeneity, susceptibility to *Plasmodium* infection and insecticide resistance (Coluzzii et al., 1979; Riehle et al., 2017; Weetman et al., 2018). Typically, we can only detect these inversions through molecular PCR‐based assays (of which many do not exist for the range of inversions karyotyped by compkaryo) or laborious and technically challenging cytological experiments (Coluzzi et al., 2002; White et al., 2007), although recent approaches using tagging SNP panels appear promising (Love et al., 2020).

We can also illuminate the putative ancestry of our samples. This is of particular interest as the two recently diverged sibling species *An. gambiae* and *An. coluzzii* may often hybridize, and have undergone extensive introgression in the recent past (Fontaine et al., 2015; Vicente et al., 2017), allowing resistance alleles to cross from one species to another (Clarkson et al., 2014; Grau‐Bové et al., 2020, 2021). Despite this, molecular assays typically target only a single marker on the X chromosome, ignoring the potential for admixture elsewhere in the genome (Caputo et al., 2021; Chabi et al., 2019; Santolamazza et al., 2008). As genome resources advance in other vectors, such as *Aedes aegypti* and *Culex pipiens*, we will expand the ancestry‐informative marker analysis of *RNA‐Seq‐Pop* to these species complexes.

### 4.2 Patterns of resistance in the Busia data set

Differential expression analysis highlighted a multitude of detoxification genes overexpressed in the selected Busia survivors, including cytochrome P450s, carboxylesterases, chemosensory proteins and ABC transporters, reflecting the broad nature of the response to insecticide exposure. Many P450 genes were ≈2‐fold overexpressed and it is not known whether this is due to constitutive differences between the strains, or induction by deltamethrin exposure in the G28 Busia strain. The *Sap2* gene in particular was highly overexpressed (10.7‐fold after deltamethrin selections and exposure), and thus serves as a strong candidate for pyrethroid resistance outside of the West African *An. coluzzii* populations in which it was originally identified (Ingham et al., 2020). *Sap2* is known to be induced upon insecticide exposure, although the relative importance of selection vs. induction by exposure cannot be determined from this experimental design.

The increase in *Vgsc*‐995S *kdr* allele frequency (previously *1014S*) following selections and segregation after exposure is as predicted given its known association with pyrethroid resistance. Interestingly, the G28 selected Busia strain showed a much stronger phenotype against permethrin than deltamethrin (Table S2A), which could partially be a result of this mutation. Earlier studies have shown a stronger protective effect of the *Vgsc*‐995S allele against permethrin than deltamethrin (Lynd et al., 2010). In agreement with this difference in *Vgsc*‐995S frequency, we find high *F*_ST_ in *Vgsc* between the G24 parental and G28 selected Busia survivors. The *Vgsc* gene is not differentially expressed between the parental Busia strain and the selected Busia survivors, meaning this result would have been missed using DEAs alone.

The 2La inversion was at much greater frequency in the G28 survivors, suggesting an association with deltamethrin resistance in Busia. Associations between the 2La inversion and insecticide resistance have been previously reported in Uganda (Weetman et al., 2018). We also find a large difference in *Cyp4J5*‐L43F mutation frequency and there is very high *F*_ST_ in this gene (0.59), which lies within the 2La inversion. Interestingly, the gene is also differentially expressed, perhaps suggesting that the 2La haplotypic background results in over‐transcription of the gene when compared to 2L+a haplotypes. It is not clear whether *Cyp4J5* is causative, or if there are other variants on the 2La haplotype(s) that are driving this shift in 2La. In agreement with this and the difference in *kdr*, we find high overall *F*_ST_ between the G24 parental and G28 Busia survivors on the 2L chromosomal arm (Table S10).

Interestingly, *RNA‐Seq‐Pop* revealed that the Kisumu reference strain exhibits a large proportion of putative *An. coluzzii* ancestry. The Kisumu reference strain was colonized from Kisumu, Kenya, in 1975 (Williams et al., 2019) from an area where *An. coluzzii* has not been recorded. The most parsimonious explanation is that the colony has been contaminated through hybridization in the insectary during its long colonization. The X chromosome is typically resistant to introgression, and consistent with a theory of a laboratory contamination event, no *An. coluzzii* variants are found on the X chromosome. The X chromosome of Kisumu also has a particularly low estimate of Watterson's Θ compared to the autosomes, which may reflect admixture present on the autosomes (Table S7A). In addition, we also find that the Kisumu strain contains the *Gste2*‐114T mutation at high frequency. In agreement with this finding, recent data show occasional low‐level resistance to DDT in this strain (Williams et al., 2019). We also observe some putative *An. coluzzii* alleles in the two Busia strains. Whilst we cannot rule out other explanations, this set of ancestry‐informative markers were derived from Mali, and therefore it is likely that some may not be truly informative of ancestry outside of this population.

In this study, we exposed the G28 selected strain in order to maximize the resistance phenotype and strengthen the genotype–phenotype association. This design choice, however, may mean estimates of allele and karyotype frequencies are overestimates and not necessarily reflective of either the unexposed G28 or G29 Busia strains. This is because susceptible G28 mosquitoes have not survived, and G28 survivors have probably already mated, meaning susceptible alleles may be passed on to the next generation, affecting allele frequencies. Equally, insecticide survivors may not go on to produce offspring. In general, we recommend sequencing appropriate controls where possible—for example, in our case, including a G28 Busia unexposed group.

When analysing RNA‐Seq data we only have read coverage in expressed parts of the genome, primarily in exons, and so we can only call genetic variants in these regions. Although not ideal, given that we expect the majority of functional variants to exist in expressed regions of the genome (Choi et al., 2009), this is not a necessarily a major drawback. Indeed, this is the premise of exome sequencing, in which only protein‐coding parts of the genome are targeted to sequence. Additionally, estimated population allele frequencies derived from RNA‐Seq data may not accurately reflect DNA‐based allele frequencies. Allele‐specific expression is one cause of this, where two or more alleles in a diploid or polyploid may be expressed at different levels, causing an imbalance. Despite this, previous studies have shown a strong correlation between expressed and true allele frequencies, particularly at higher sequencing depth (Jehl et al., 2021; Lopez‐Maestre et al., 2016; Oikkonen & Lise, 2017; Quinn et al., 2013). In this study, we performed RNASeq at a high sequencing depth, and therefore can have more confidence overall in our genotype calls and subsequent analyses. We recommend generally that for DEAs, low‐coverage RNA‐Seq is sufficient (10–25 million reads, or 5–13.5× coverage for *An. gambiae*), with a higher number of biological replicates (four or more). For variant analyses, higher coverage is preferred (25–60 million reads, or 13.5–32.4× coverage for *An. gambiae*), with a high number of individuals pooled per replicate (≥10).

Although other studies present strategies to call variants from RNA‐Seq data (Brouard & Bissonnette, 2022; Jehl et al., 2021; Piskol et al., 2013; Quinn et al., 2013), none of these studies present convenient, reproducible bioinformatic pipelines to implement their suggested strategies, instead requiring the user to manually perform each step. In addition to that, we found no studies that present pipelines to call variants and also perform analyses on the resulting SNP data. Although a previous study showed that population genomic signals can be extracted from RNA‐Seq data, they did not package their analysis into any usable workflow, and the analyses themselves are limited in comparison to *RNA‐Seq‐Pop* (Thorstensen et al., 2020). Based on the lack of comparable, easy‐to‐use workflows, we envisage that *RNA‐Seq‐Pop* will prove useful to many researchers in a range of biological fields.

## AUTHOR CONTRIBUTIONS

SCN, DW and MJD conceived and designed the study. SCN and AO performed all experiments and SCN analysed the data. SCN, DW and MJD drafted the manuscript, and all authors read and approved the final version.

## CONFLICT OF INTEREST

We declare no conflict of interests.

## Supporting information


Data S1

Data S2

Data S3

Supplementary file

## Data Availability

The workflow is hosted at https://github.com/sanjaynagi/rna‐seq‐pop. We welcome and encourage any feedback or contributions to *RNA‐Seq‐Pop*. The variant of interest file is versioned and is included in the GitHub repository. Raw sequence data are deposited at the ENA under BioProject PRJNA748581. The modified version of compkaryo is found at https://github.com/sanjaynagi/compkaryo.
