# Supplementary material for: RNA‐Seq‐Pop: Exploiting the sequence in RNA sequencing—A Snakemake workflow reveals patterns of insecticide resistance in the malaria vector Anopheles gambiae
Source: Mol Ecol Resour. 2023 Feb 10;23(4):946–61. doi: 10.1111/1755-0998.13759 (PMC10568660; doi:10.1111/1755-0998.13759)

COE, pfam domains = COesterase

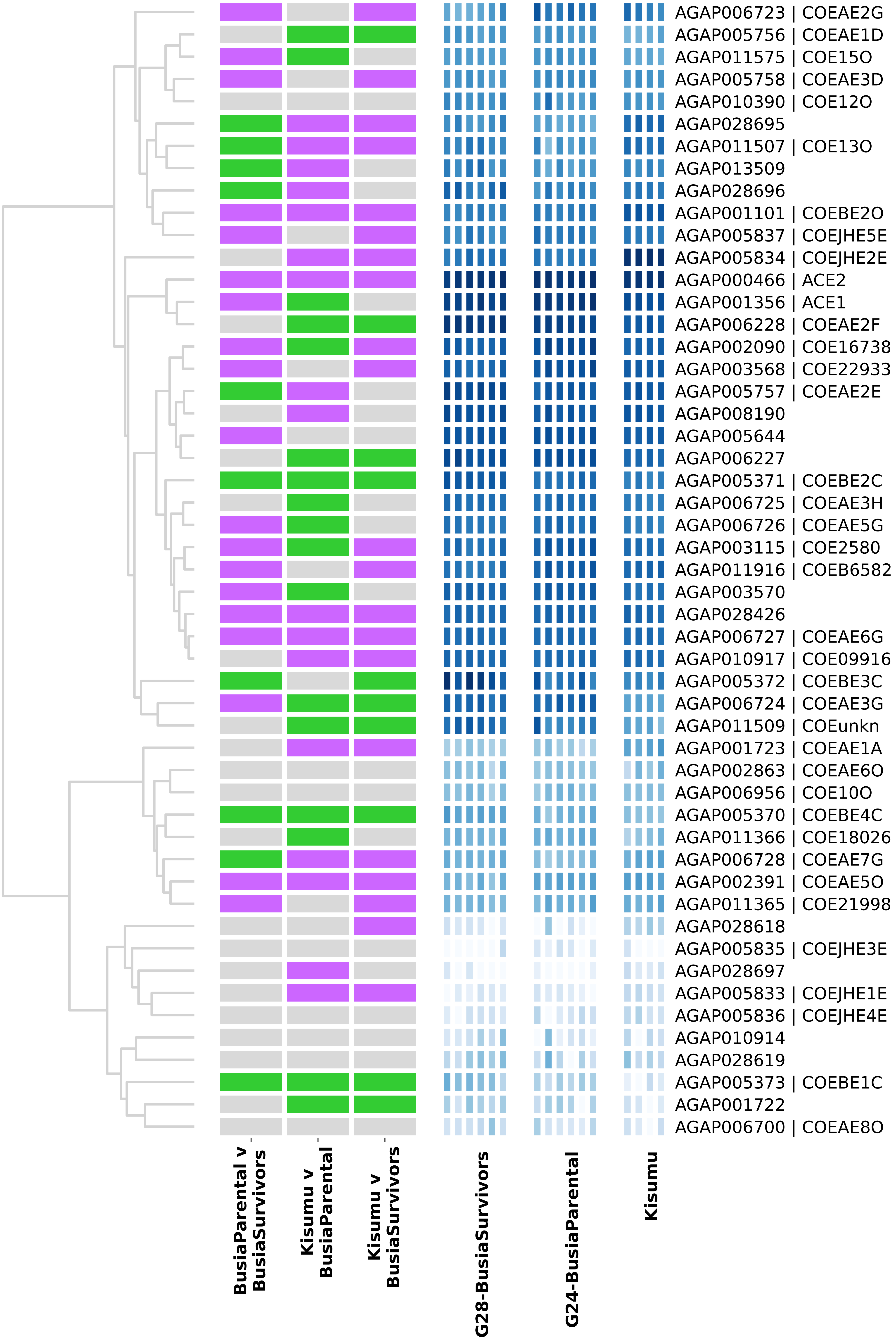

OBP, pfam domains = PBP_GOBP

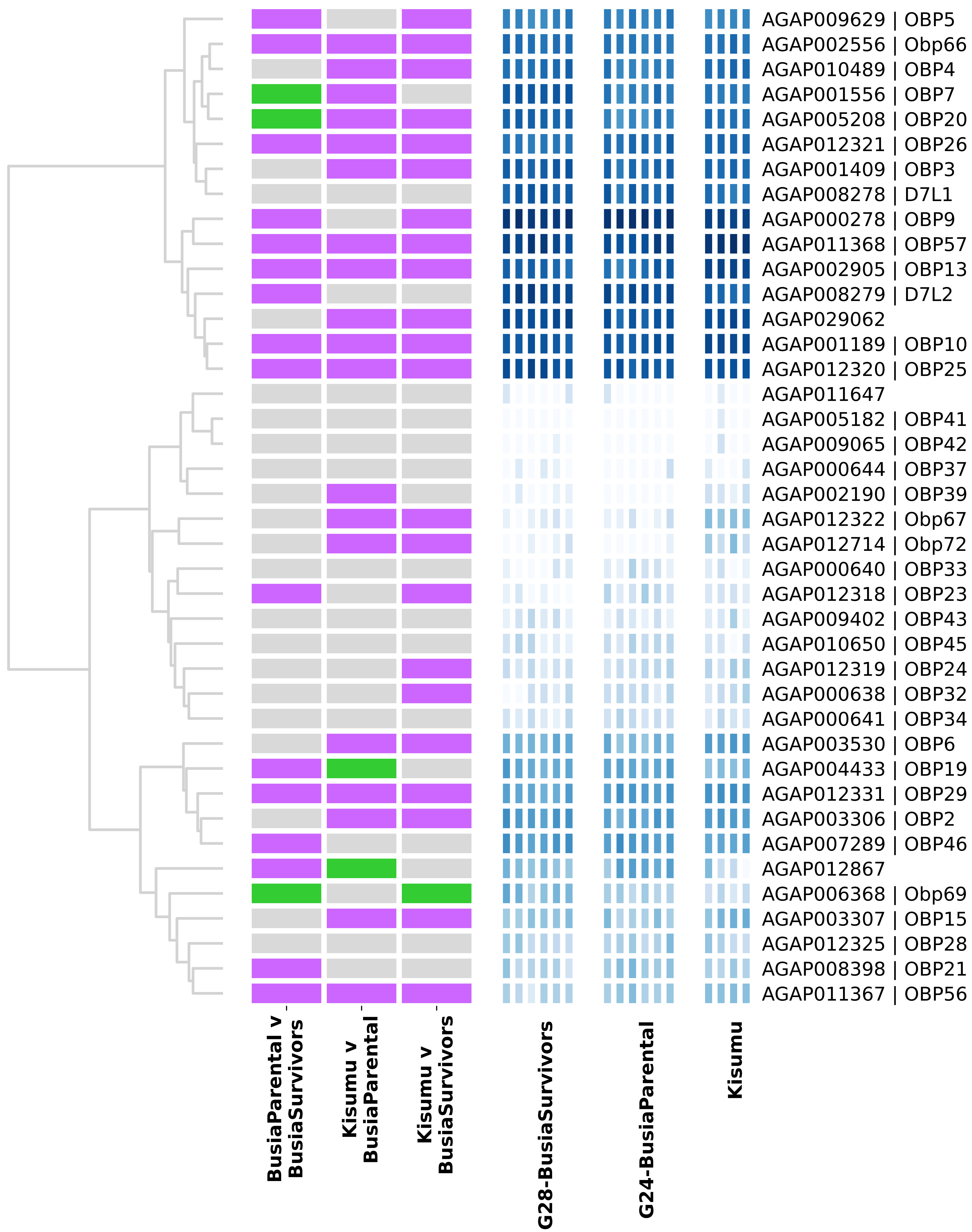

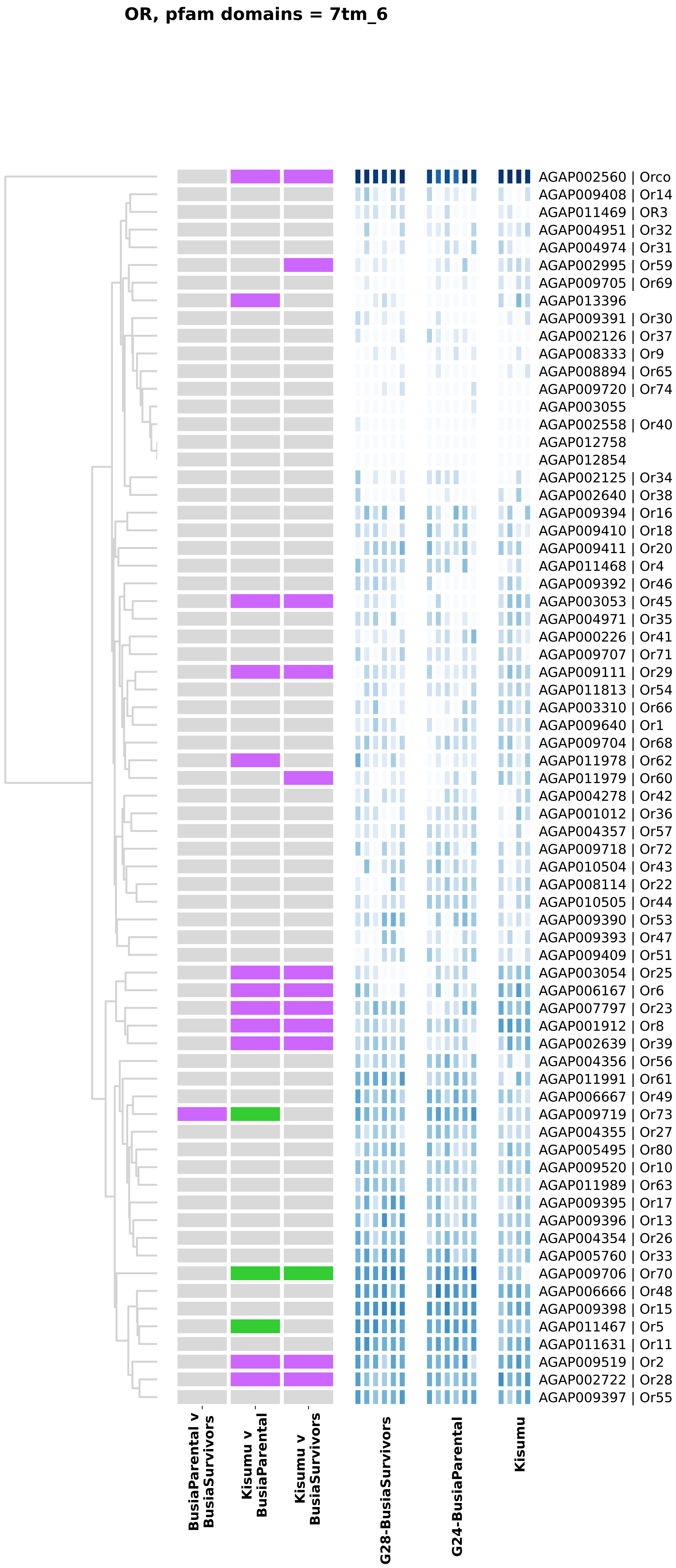

OR, pfam domains = 7tm_6
AGAP002560 | Orco
AGAP009408 | Or14
AGAP011469 | OR3
AGAP004951 | Or32
AGAP004974 | Or31
AGAP002995 | Or59
AGAP009705 | Or69
AGAP013396
AGAP009391 | Or30
AGAP002126 | Or37
AGAP008333 | Or9
AGAP008894 | Or65
AGAP009720 | Or74
AGAP003055
AGAP002558 | Or40
AGAP012758
AGAP012854
AGAP002125 | Or34
AGAP002640 | Or38
AGAP009394 | Or16
AGAP009410 | Or18
AGAP009411 | Or20
AGAP011468 | Or4
AGAP009392 | Or46
AGAP003053 | Or45
AGAP004971 | Or35
AGAP000226 | Or41
AGAP009707 | Or71
AGAP009111 | Or29
AGAP011813 | Or54
AGAP003310 | Or66
AGAP009640 | Or1
AGAP009704 | Or68
AGAP011978 | Or62
AGAP011979 | Or60
AGAP004278 | Or42
AGAP001012 | Or36
AGAP004357 | Or57
AGAP009718 | Or72
AGAP010504 | Or43
AGAP008114 | Or22
AGAP010505 | Or44
AGAP009390 | Or53
AGAP009393 | Or47
AGAP009409 | Or51
AGAP003054 | Or25
AGAP006167 | Or6
AGAP007797 | Or23
AGAP001912 | Or8
AGAP002639 | Or39
AGAP004356 | Or56
AGAP011991 | Or61
AGAP006667 | Or49
AGAP009719 | Or73
AGAP004355 | Or27
AGAP005495 | Or80
AGAP009520 | Or10
AGAP011989 | Or63
AGAP009395 | Or17
AGAP009396 | Or13
AGAP004354 | Or26
AGAP005760 | Or33
AGAP009706 | Or70
AGAP006666 | Or48
AGAP009398 | Or15
AGAP011467 | Or5
AGAP011631 | Or11
AGAP009519 | Or2
AGAP002722 | Or28
AGAP009397 | Or55
BusiaParental v BusiaSurvivors
Kisumu v BusiaParental
Kisumu v BusiaSurvivors
G28-BusiaSurvivors
G24-BusiaParental
Kisumu

Io, pfam domains = ['Lig_chan', '7tm_1']

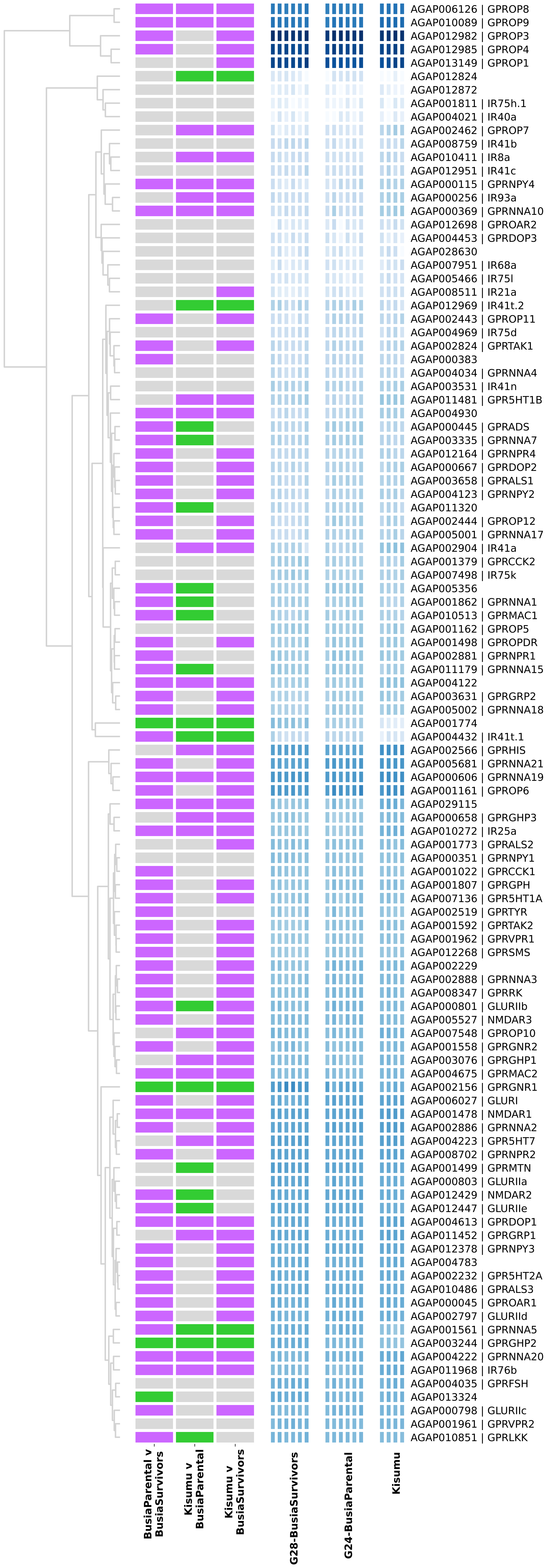

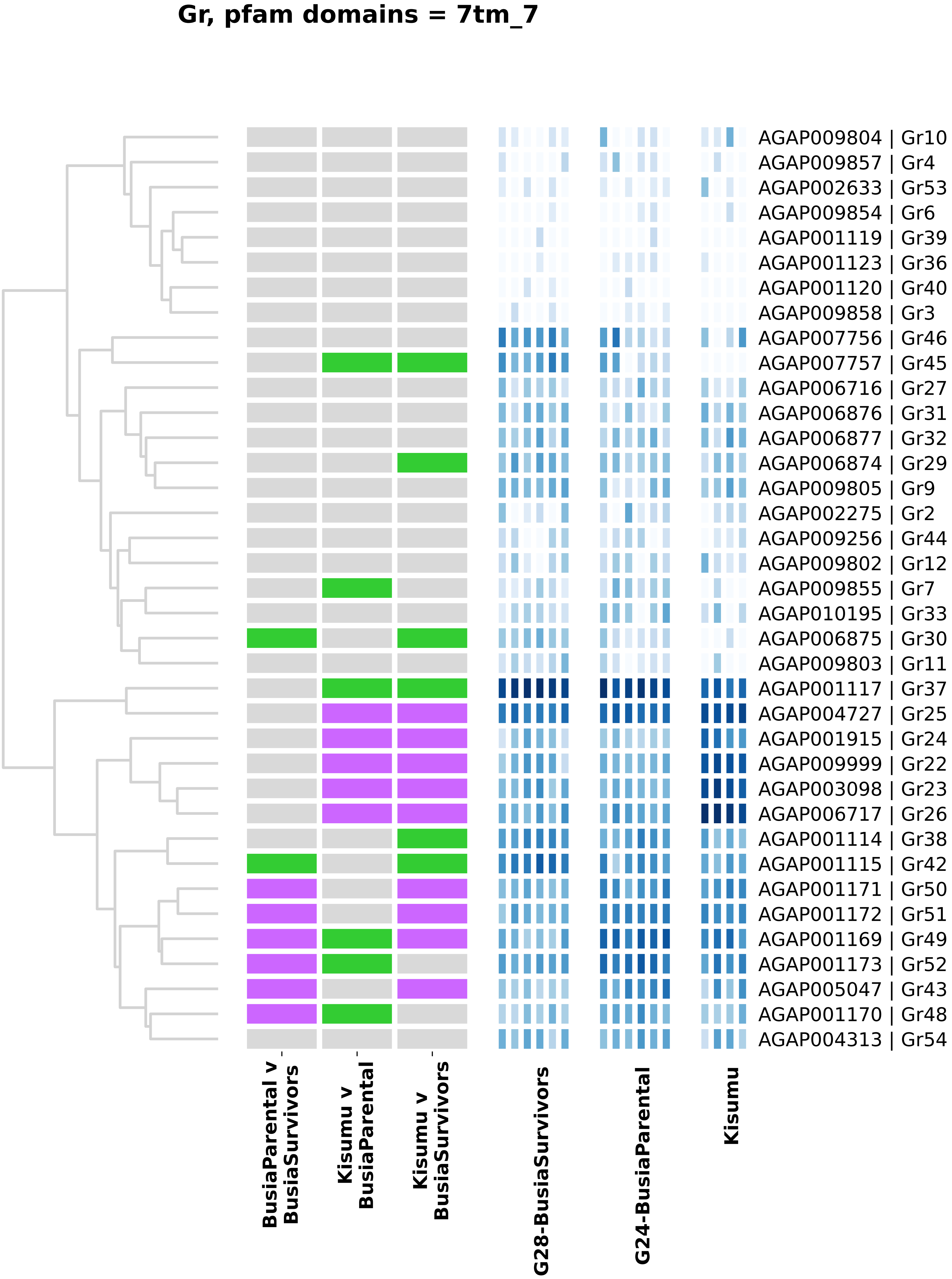

Gr, pfam domains = 7tm_7
AGAP009804 | Gr10
AGAP009857 | Gr4
AGAP002633 | Gr53
AGAP009854 | Gr6
AGAP001119 | Gr39
AGAP001123 | Gr36
AGAP001120 | Gr40
AGAP009858 | Gr3
AGAP007756 | Gr46
AGAP007757 | Gr45
AGAP006716 | Gr27
AGAP006876 | Gr31
AGAP006877 | Gr32
AGAP006874 | Gr29
AGAP009805 | Gr9
AGAP002275 | Gr2
AGAP009256 | Gr44
AGAP009802 | Gr12
AGAP009855 | Gr7
AGAP010195 | Gr33
AGAP006875 | Gr30
AGAP009803 | Gr11
AGAP001117 | Gr37
AGAP004727 | Gr25
AGAP001915 | Gr24
AGAP009999 | Gr22
AGAP003098 | Gr23
AGAP006717 | Gr26
AGAP001114 | Gr38
AGAP001115 | Gr42
AGAP001171 | Gr50
AGAP001172 | Gr51
AGAP001169 | Gr49
AGAP001173 | Gr52
AGAP005047 | Gr43
AGAP001170 | Gr48
AGAP004313 | Gr54
BusiaParental v BusiaSurvivors
Kisumu v BusiaParental
Kisumu v BusiaSurvivors
G28-BusiaSurvivors
G24-BusiaParental
Kisumu

P450, pfam domains = p450

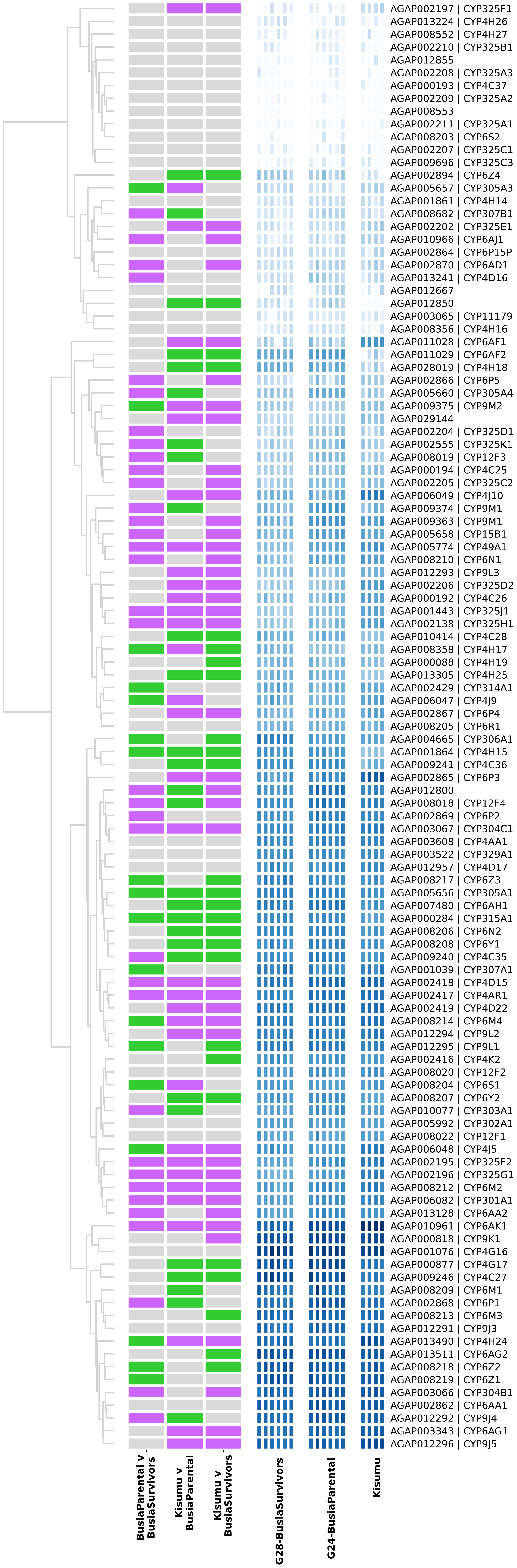

Gst, pfam domains = ['GST_N', 'GST_N_3', 'GST_C']

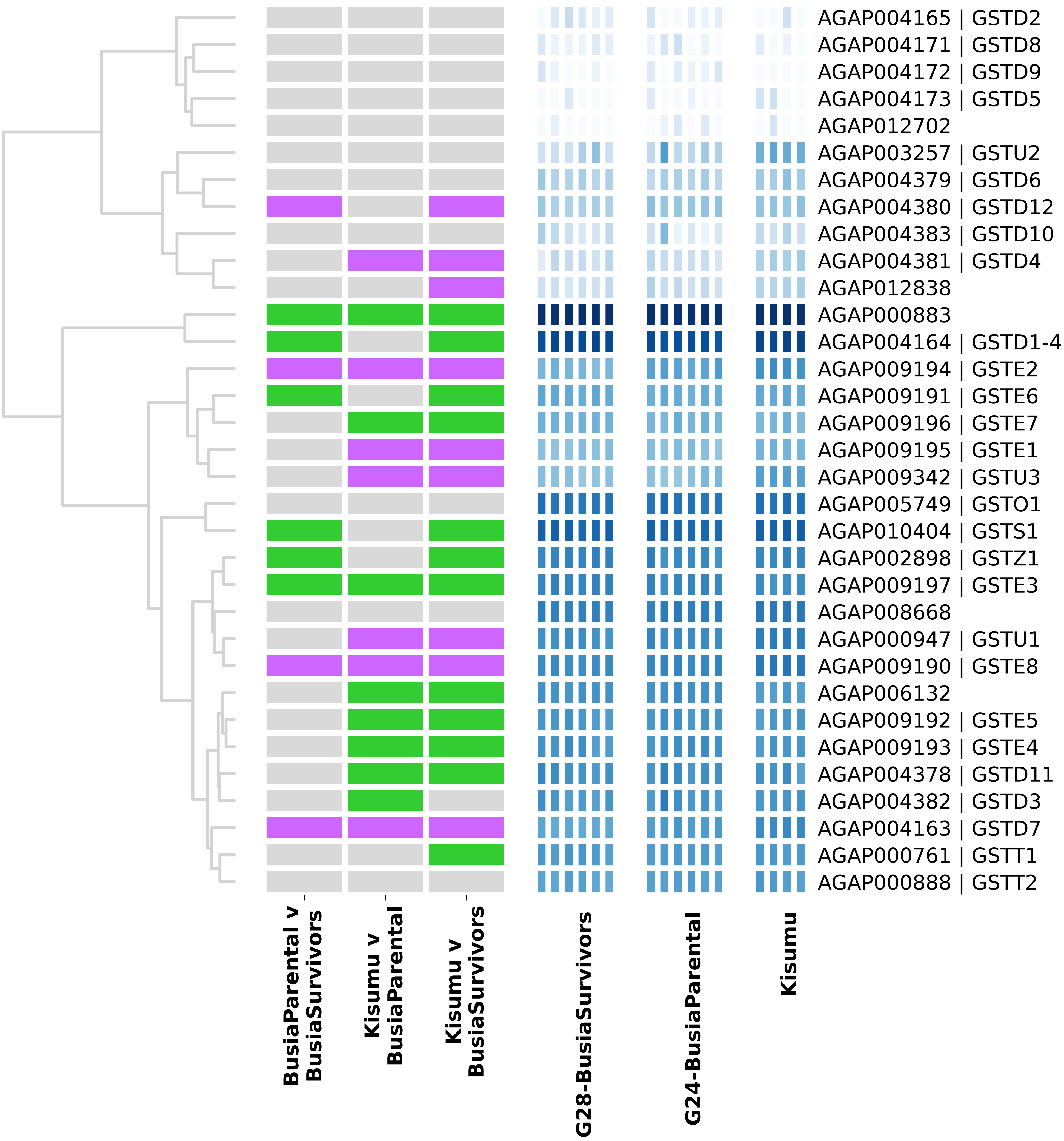

ABC, pfam domains = ['ABC_membrane', 'ABC_tran']

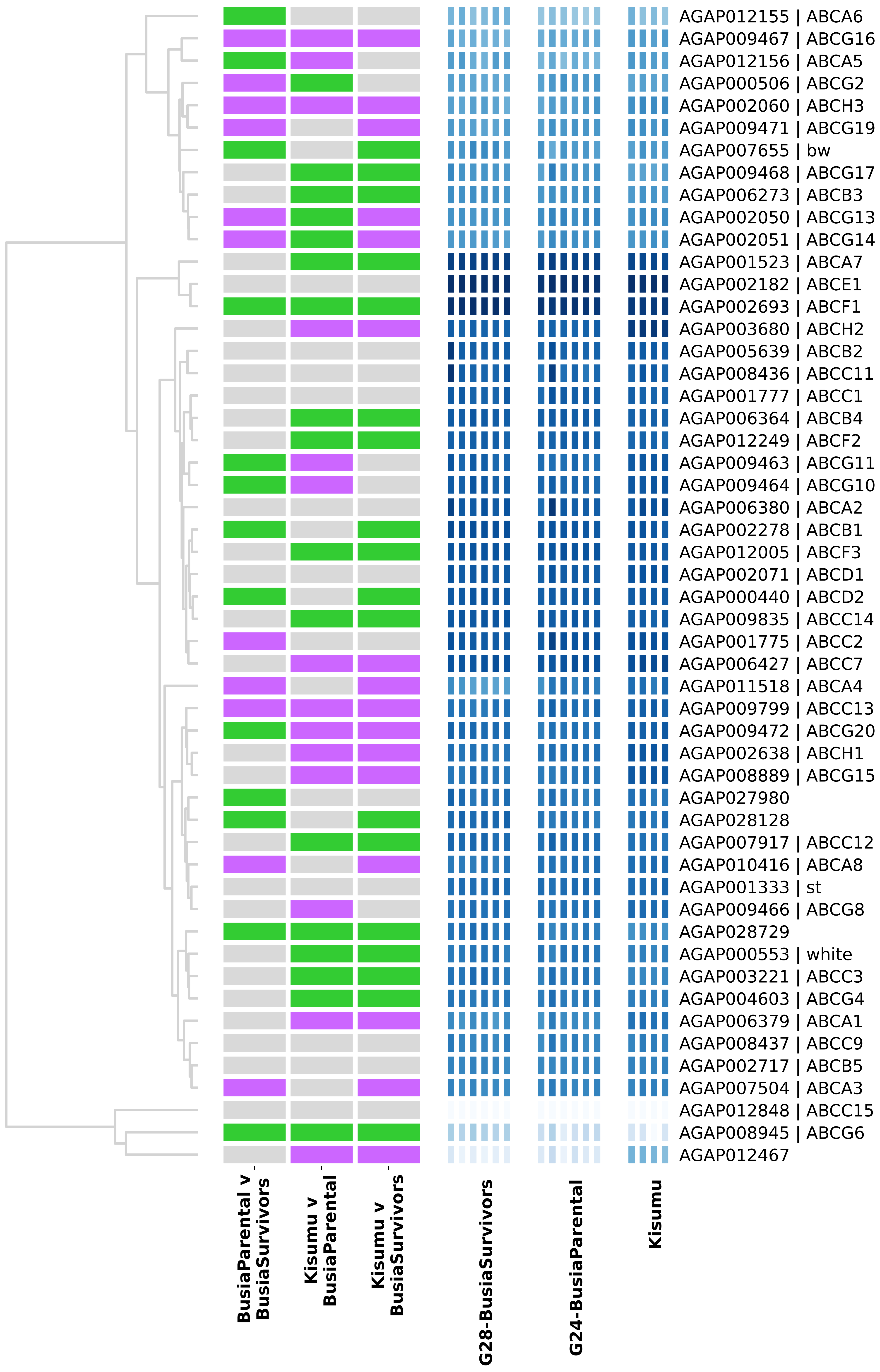

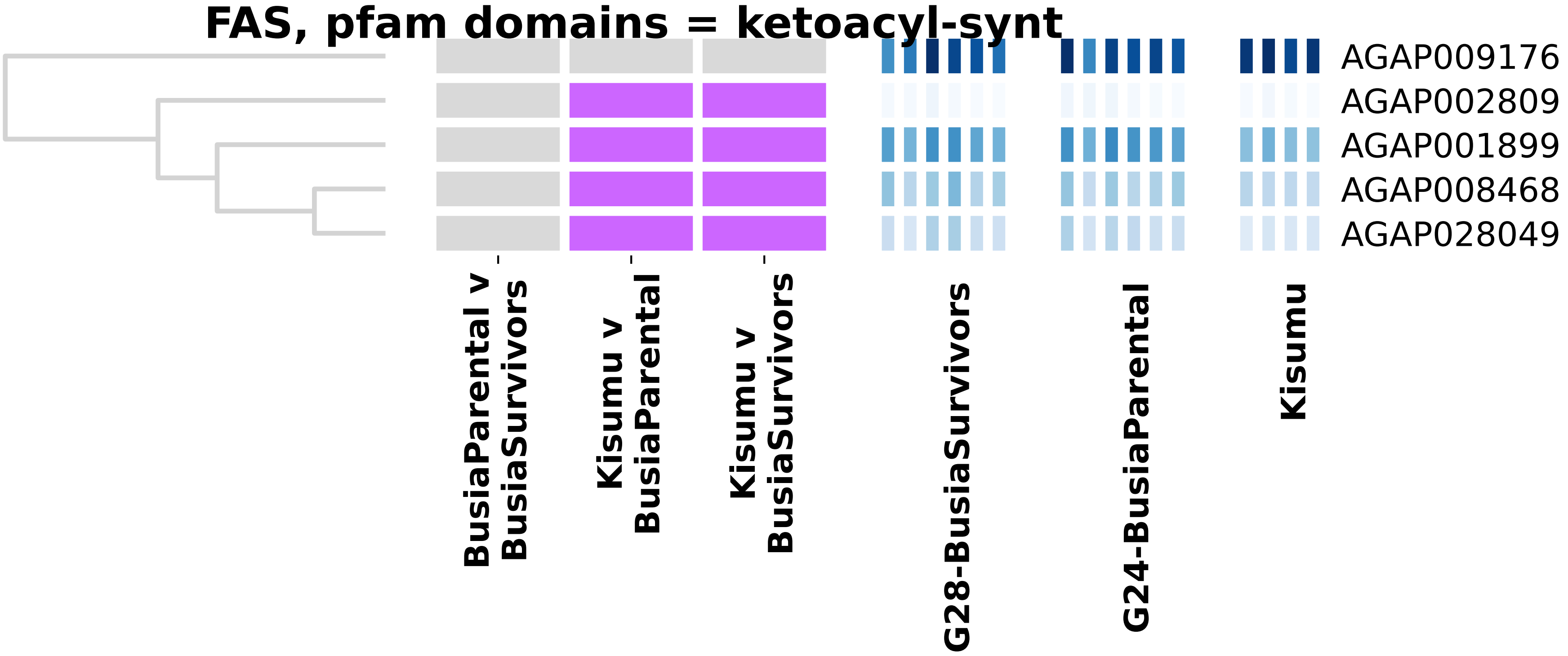

FAS, pfam domains = ketoacyl-synt
AGAP009176
AGAP002809
AGAP001899
AGAP008468
AGAP028049
BusiaParental v BusiaSurvivors
Kisumu v BusiaParental
Kisumu v BusiaSurvivors
G28-BusiaSurvivors
G24-BusiaParental
Kisumu

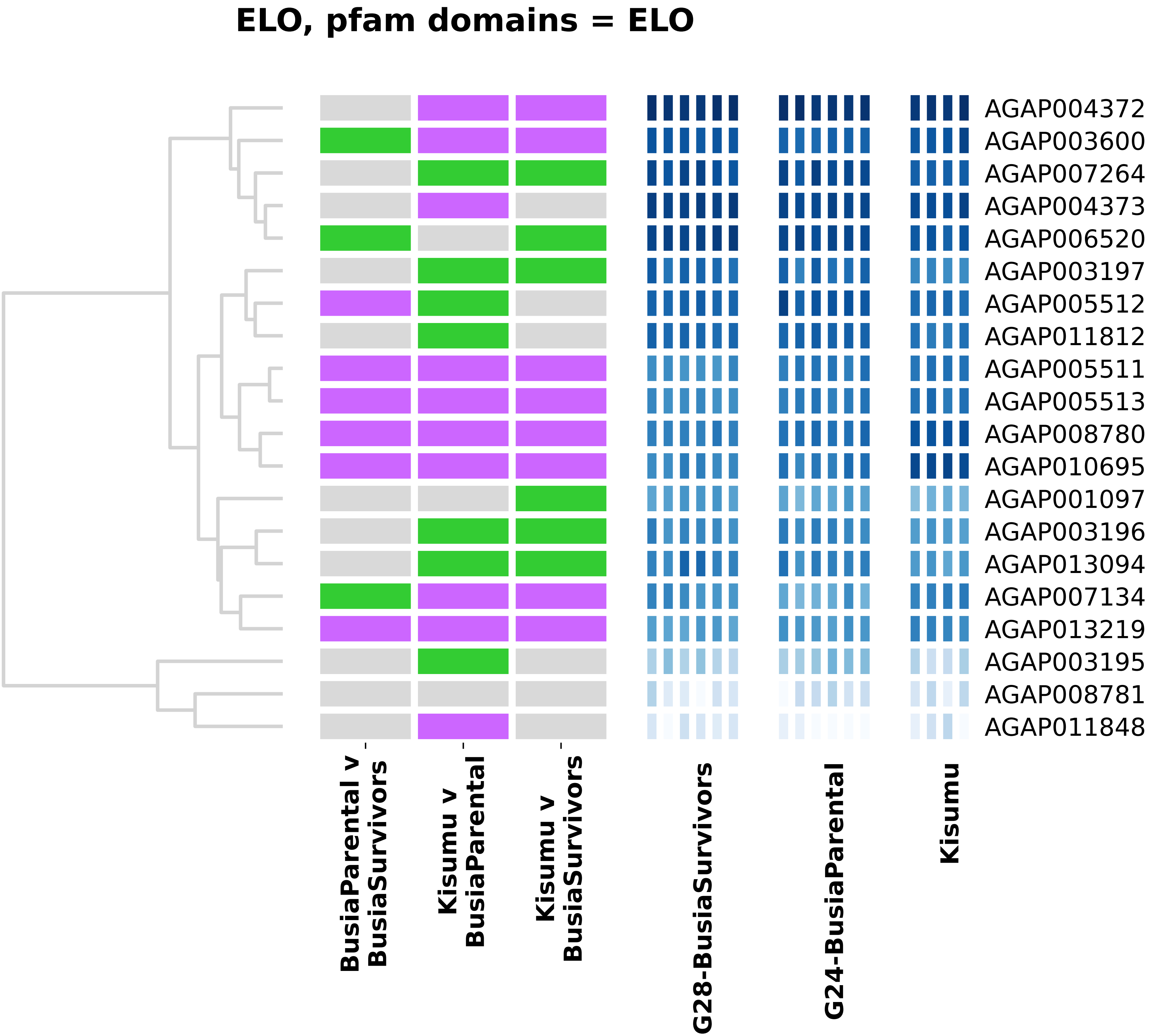
ELO, pfam domains = ELO
AGAP004372
AGAP003600
AGAP007264
AGAP004373
AGAP006520
AGAP003197
AGAP005512
AGAP011812
AGAP005511
AGAP005513
AGAP008780
AGAP010695
AGAP001097
AGAP003196
AGAP013094
AGAP007134
AGAP013219
AGAP003195
AGAP008781
AGAP011848
BusiaParental v BusiaSurvivors
Kisumu v BusiaParental
Kisumu v BusiaSurvivors
G28-BusiaSurvivors
G24-BusiaParental
Kisumu

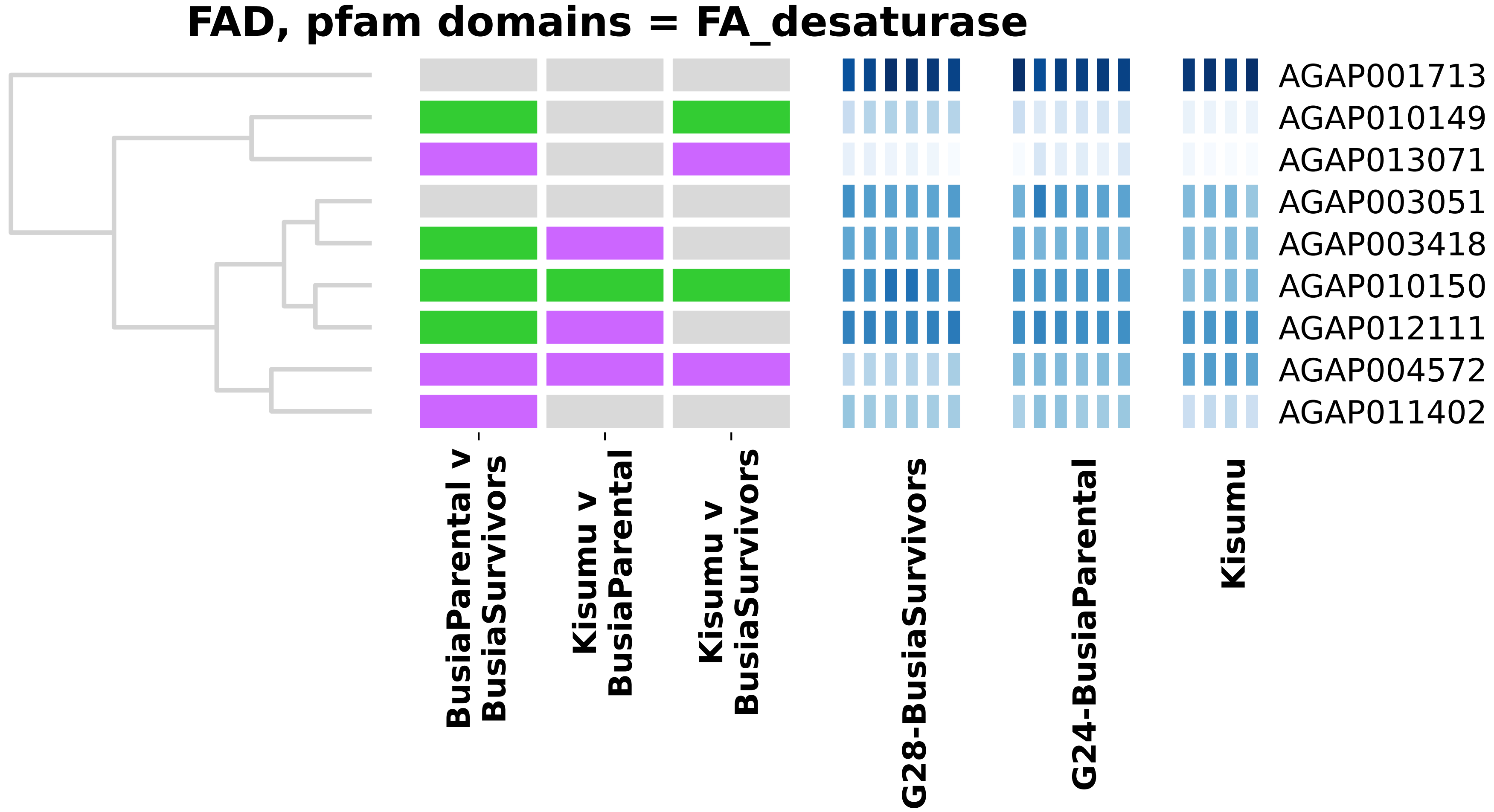
FAD, pfam domains = FA_desaturase
AGAP001713
AGAP010149
AGAP013071
AGAP003051
AGAP003418
AGAP010150
AGAP012111
AGAP004572
AGAP011402
BusiaParental v BusiaSurvivors
Kisumu v BusiaParental
Kisumu v BusiaSurvivors
G28-BusiaSurvivors
G24-BusiaParental
Kisumu

FAR, pfam domains = NAD_binding_4

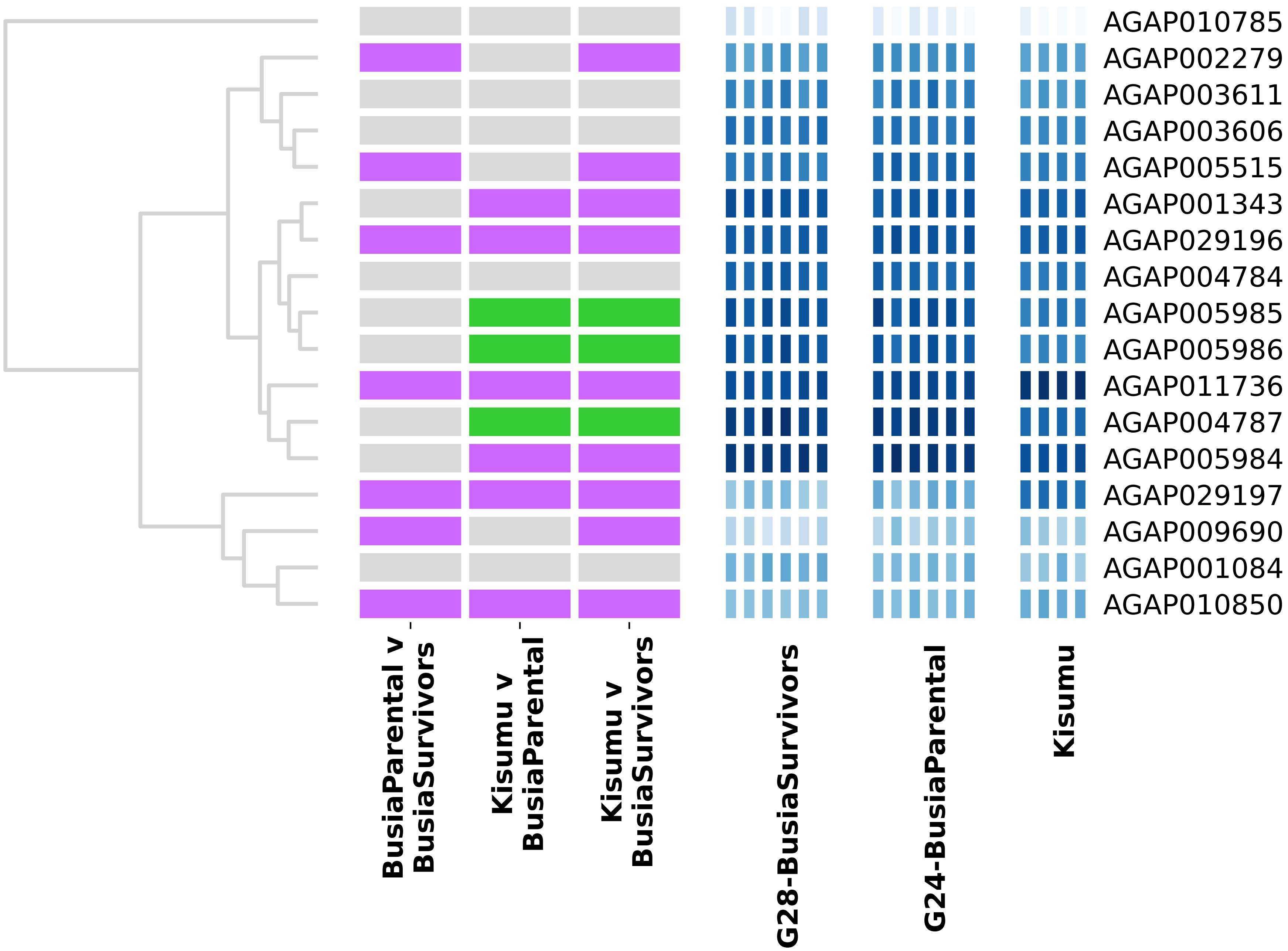

Supplement: Supplementary file 3 — Data S3 [file MEN-23-946-s004.pdf]
