# Supplementary material for: RNA‐Seq‐Pop: Exploiting the sequence in RNA sequencing—A Snakemake workflow reveals patterns of insecticide resistance in the malaria vector Anopheles gambiae
Source: Mol Ecol Resour. 2023 Feb 10;23(4):946–61. doi: 10.1111/1755-0998.13759 (PMC10568660; doi:10.1111/1755-0998.13759)
Supplement: Supplementary file 4 — Supplementary file [file MEN-23-946-s002.pdf]

Supplementary – Nagi *et al.*, 2023

1) Literature review – published studies of RNA-Sequencing in disease vectors

Databases – Web of Science
Date - 03/05/2022
Search terms –

1) (Anopheles OR Aedes OR Culex OR vector OR tsetse OR sandfly) AND (rna-seq OR rna-sequencing OR rna seq OR expression OR transcriptomics)
2) Mosquito AND (rna-seq OR rna-sequencing OR rna seq OR expression OR transcriptomics)

Title	Citation	Taxon	Phenotype / purpose	Year	Sequence data utilised
The RNA-Seq approach to studying the expression of mosquito mitochondrial genes	(Neira-Oviedo et al., 2011)	*Aedes aegypti, Anopheles gambiae, and Anopheles quadrimaculatus*	Mitochondria	2011	
RNA-seq analyses of blood-induced changes in gene expression in the mosquito vector species, *Aedes aegypti*	(Bonizzoni et al., 2011)	*Aedes aegypti*	Bloodmeal	2011	
Comparative Transcriptome Analyses of Deltamethrin-Resistant and -Susceptible *Anopheles gambiae* Mosquitoes from Kenya by RNA-Seq	(Bonizzoni et al., 2012)	*Anopheles gambiae*	Insecticide resistance (pyrethroid)	2012	
RNA-Seq reveals early distinctions and late convergence of gene expression between diapause and quiescence in the Asian tiger mosquito, *Aedes albopictus*	(Poelchau et al., 2013)	*Aedes albopictus*	Diapause	2013	
The Developmental Transcriptome of the Mosquito *Aedes aegypt*i, an Invasive Species and Major Arbovirus Vector	(Akbari et al., 2013)	*Aedes aegypti*	Life-stage	2013	
De novo transcriptome sequencing and sequence analysis of the malaria vector *Anopheles sinensis*	(Chen et al., 2014)	*Anopheles sinensis*	Annotation	2014	
Comparative analysis of response to selection with three insecticides in the dengue mosquito *Aedes aegypti* using mRNA sequencing	(David et al., 2014)	*Aedes aegypti*	Insecticide resistance (permethrin, imidacloprid, propoxur)	2014	Yes
Dual RNA-seq of Parasite and Host Reveals Gene Expression Dynamics during Filarial Worm–Mosquito Interactions	(Choi et al., 2014)	*Aedes aegypti*	Host-parasite	2014	
RNA-seq analyses of changes in the *Anopheles gambiae* transcriptome associated with resistance to pyrethroids in Kenya: identification of candidate-resistance genes and candidate-resistance SNPs	(Bonizzoni et al., 2015)	*Anopheles gambiae*	Insecticide resistance (pyrethroid)	2015	Yes
Comparative transcriptome analyses of deltamethrin-susceptible and -resistant *Culex pipiens pallens* by RNA-seq	(Lv et al., 2016)	*Culex pipiens*	Insecticide resistance (pyrethroid)	2016	
Single molecule RNA sequencing uncovers trans-splicing and improves annotations in *Anopheles stephensi*	(Jiang et al., 2017)	*Anopheles stephensi*	Annotation	2017	
Comparative Transcriptomics of Malaria Mosquito Testes: Function, Evolution, and Linkage	(Cassone et al., 2017)	*A. gambiae and A. merus*	Spermatogenesis	2017	
In the hunt for genomic markers of metabolic resistance to pyrethroids in the mosquito *Aedes aegypti*: An integrated next-generation sequencing approach	(Faucon et al., 2017)	*Aedes aegypti*	Insecticide resistance (pyrethroid)	2017	Yes
RNA-Seq Comparison of Larval and Adult Malpighian Tubules of the Yellow Fever Mosquito *Aedes aegypt*i Reveals Life Stage-Specific Changes in Renal Function	(Li et al., 2017)	*Aedes aegypti*	Life-stage	2017	
The choreography of the chemical defensome response to insecticide stress: insights into the *Anopheles stephensi* transcriptome using RNA-Seq	(De Marco et al., 2017)	*Anopheles stephensi*	Insecticide resistance	2017	
Blood-induced differential gene expression in *Anopheles dirus* evaluated using RNA sequencing	(Mongkol et al., 2018)	*Anopheles dirus*	Bloodmeal	2018	
High-resolution transcriptional profiling of *Anopheles gambiae* spermatogenesis reveals mechanisms of sex chromosome regulation	(Taxiarchi et al., 2019)	*Anopheles gambiae*	Spermatogenesis	2019	
Transcriptome Sequencing and Analysis of Changes Associated with Insecticide Resistance in the Dengue Mosquito (*Aedes aegypti*) in Vietnam	(Lien et al., 2019)	*Aedes aegypti*	Insecticide resistance	2019	
Genome-wide gene expression profiling reveals that cuticle alterations and P450 detoxification are associated with deltamethrin and DDT resistance in *Anopheles arabiensis* populations from Ethiopia	(Simma et al., 2019)	*Anopheles arabiensis*	Insecticide resistance (pyrethroid)	2019	
UDP-glycosyltransferase genes and their association and mutations associated with	(Zhou et al., 2019)	*Anopheles sinensis*	Insecticide resistance	2019	

pyrethroid resistance in Anopheles sinensis (Diptera: Culicidae)			(pyrethroid)		
RNASeq Analysis of *Aedes albopictus* Mosquito Midguts after Chikungunya Virus Infection	(Vedururu et al., 2019)	*Aedes albopictus*	Host-parasite	2019	
Contrasting patterns of gene expression indicate differing pyrethroid resistance mechanisms across the range of the New World malaria vector *Anopheles albimanus*	(Mackenzie-Impoinvil et al., 2019)	*Anopheles albimanus*	Insecticide resistance (pyrethroid)	2019	
Transcriptome analysis of *Anopheles dirus* and *Plasmodium vivax* at ookinete and oocyst stages	(Boonkaew et al., 2020)	*Anopheles dirus*	Plasmodium infection	2020	
Transcript Assembly and Quantification by RNA-Seq Reveals Significant Differences in Gene Expression and Genetic Variants in Mosquitoes of the *Culex pipiens* (Diptera: Culicidae) Complex	(Kang et al., 2021)	*Culex pipiens*	Insecticide resistance (pyrethroid)	2021	Yes
Integration of whole genome sequencing and transcriptomics reveals a complex picture of the reestablishment of insecticide resistance in the major malaria vector *Anopheles coluzzii*	(Ingham et al., 2021)	*Anopheles gambiae*	Insecticide resistance (pyrethroid)	2021	
Transcriptome comparison of dengue-susceptible and -resistant field derived strains of Colombian *Aedes aegypti* using RNA-sequencing	(Coatsworth et al., 2021)	*Aedes aegypti*	Vector competence	2021	
Transcriptomic and proteomic analysis of pyrethroid resistance in the CKR strain of *Aedes aegypti*	(Sun et al., 2021)	*Aedes aegypti*	Insecticide resistance (pyrethroid)	2021	
RNA-Seq analysis of blood meal induced gene-expression changes in *Aedes aegypti* ovaries	(Nag et al., 2021)	*Aedes aegypti*	Bloodmeal	2021	
Sympatric Populations of the *Anopheles gambiae* Complex in Southwest Burkina Faso Evolve Multiple Diverse Resistance Mechanisms in Response to Intense Selection Pressure with Pyrethroids	(Williams et al., 2022)	*Anopheles gambiae*	Insecticide resistance (pyrethroid)	2022	
RNAseq-based gene expression profiling of the *Anopheles funestus* pyrethroid-resistant strain FUMOZ highlights the predominant role of the duplicated CYP6P9a/b cytochrome P450s	(Wondji et al., 2022)	*Anopheles funestus*	Insecticide resistance (pyrethroid)	2022	
Transcriptome profiling reveals sex-specific gene expressions in pupal and adult stages of the mosquito *Culex pipiens*	(Martynova et al., 2022)	*Culex pipiens*	Life-stage	2022	
A whole transcriptomic approach provides novel insights into the molecular basis of organophosphate and pyrethroid resistance in *Anopheles arabiensis* from Ethiopia	(Messenger et al., 2021)	*Anopheles arabiensis*	Insecticide resistance	2022	Yes

2) Colony selection regime

Blood fed *Anopheles gambiae s.s* were collected from Busia, Uganda, in November 2018, using a prokopack aspirator. Approximately 200 mosquitoes were collected from 12 different homes in the village of South-Bugwere. Eggs were transported to the Liverpool School of Tropical Medicine and mosquitoes were reared in the insectaries at approximately 75% RH and 27°C, with a 12:12 hour light:dark photoperiod.

Between generations 10 and 24, due to the COVID-19 pandemic, Busia mosquitoes were not selected against deltamethrin to maintain their insecticide resistance status. As a result, the colony had lost resistance by G24, displaying 100% mortality to a 1 hour 0.05% deltamethrin (1X) WHO paper exposure, and 92.6% mortality to permethrin 0.75% (1X). Mosquitoes were then selected on 1X deltamethrin WHO papers for four consecutive generations (G24-G27), initially of 15 minute exposures, and subsequently for one hour.

A) Profiling

Generation	Insecticide	Dead	Total	Mortality (%)
G24	Deltamethrin	99	99	100.0
G24	Permethrin	88	95	92.6
G25	Deltamethrin	47	54	87.0
G25	Permethrin	15	47	31.9
G26	Permethrin	9	30	30.0
G27	Deltamethrin	164	217	75.6
G28	Deltamethrin	145	208	69.7
G28	Permethrin	23	106	21.7

B) Selections

1X deltamethrin (0.05%)

Generation	Exposure. (mins)	Dead	Total	Mortality (%)
G24	15	1111	1205	92.2
G25	15	348	456	76.3
G26	60	265	327	81.0
G27	60	164	217	75.6

3)

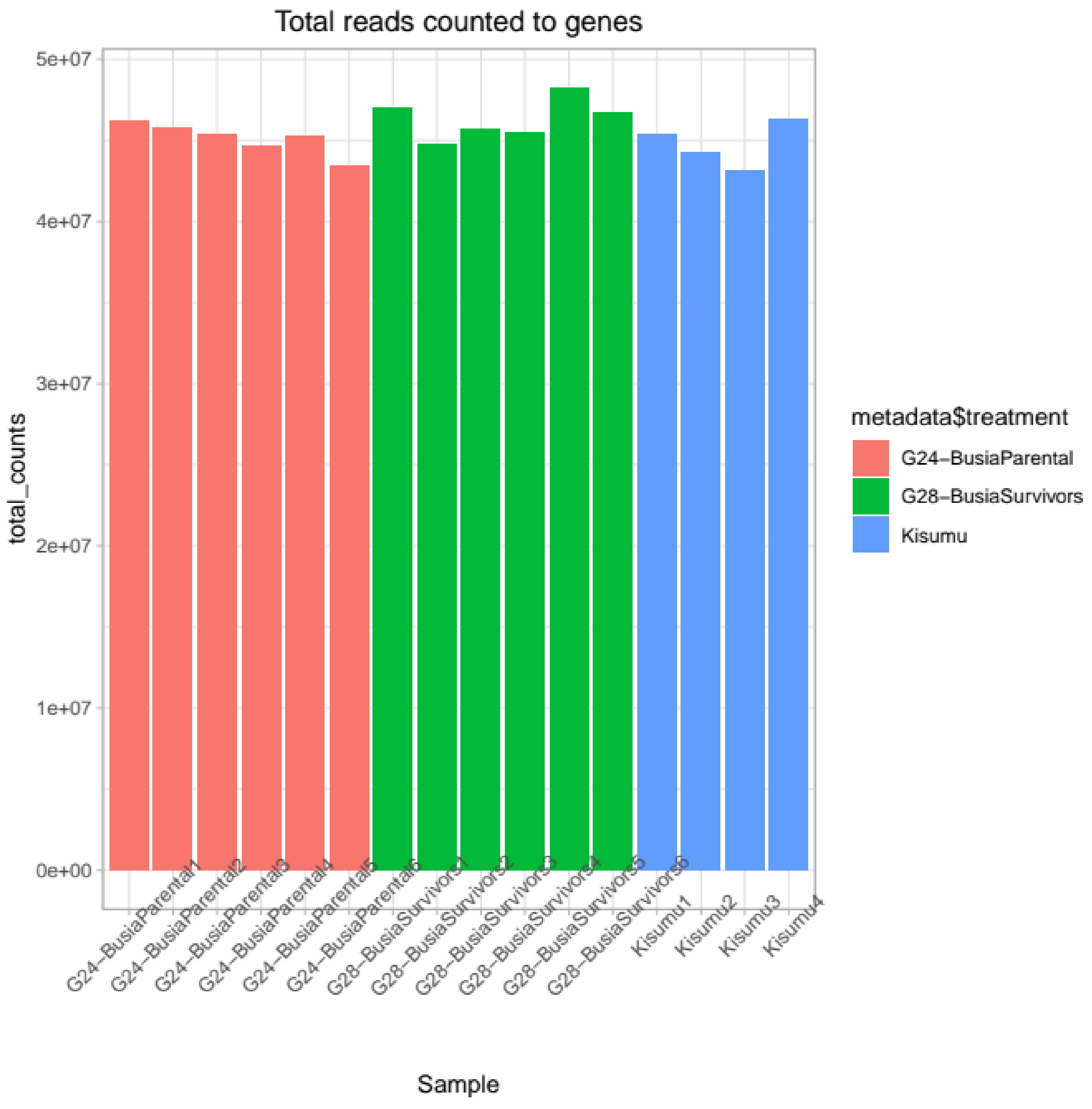
Total reads counted to genes
total_counts
5e+07
4e+07
3e+07
2e+07
1e+07
0e+00
G24–BusiaParental1
G24–BusiaParental2
G24–BusiaParental3
G24–BusiaParental4
G24–BusiaParental5
G24–BusiaParental6
G28–BusiaSurvivors1
G28–BusiaSurvivors2
G28–BusiaSurvivors3
G28–BusiaSurvivors4
G28–BusiaSurvivors5
G28–BusiaSurvivors6
Kisumu1
Kisumu2
Kisumu3
Kisumu4
Sample
metadata$treatment
G24–BusiaParental
G28–BusiaSurvivors
Kisumu

4) Volcano plots

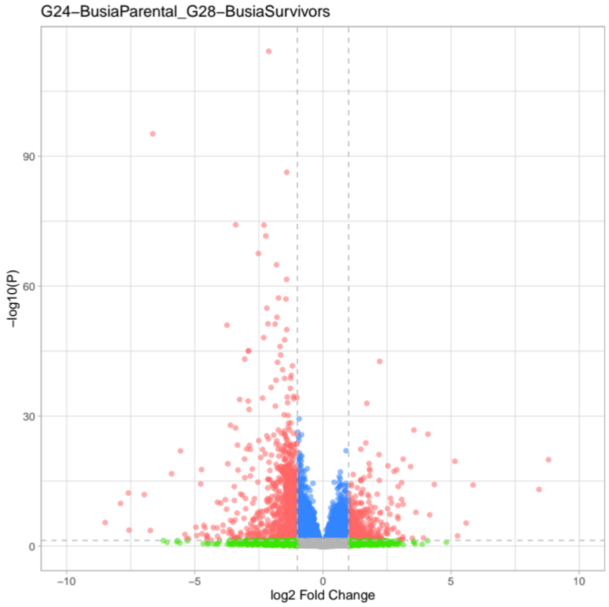

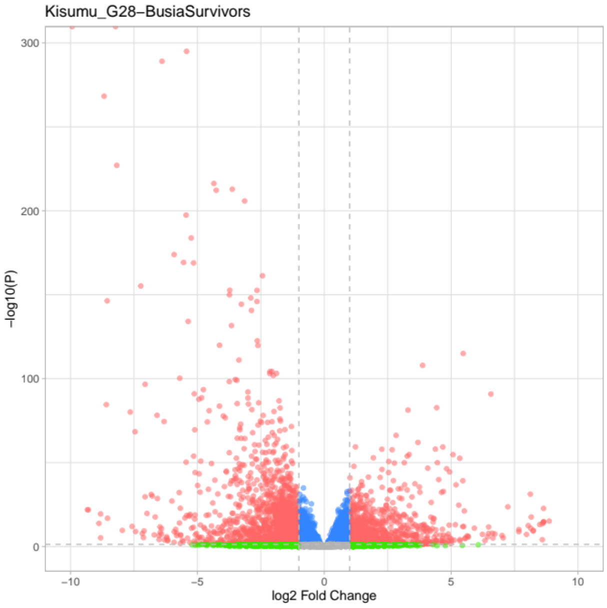

Suppl. Figure 4: A volcano plot showing gene expression differences between Busia Parental and Busia Selected survivors. -Log10 P-values are plotted against Log2 Fold Change. Red=genes with adjusted p-value < 0.05 and an absolute fold change > 2, green= adjusted pvalue > 0.05 and a fold change > 2, blue= adjusted pvalue > 0.05 and a fold change < 2. An outlier (AGAP012637) has been removed for visualisation purposes.

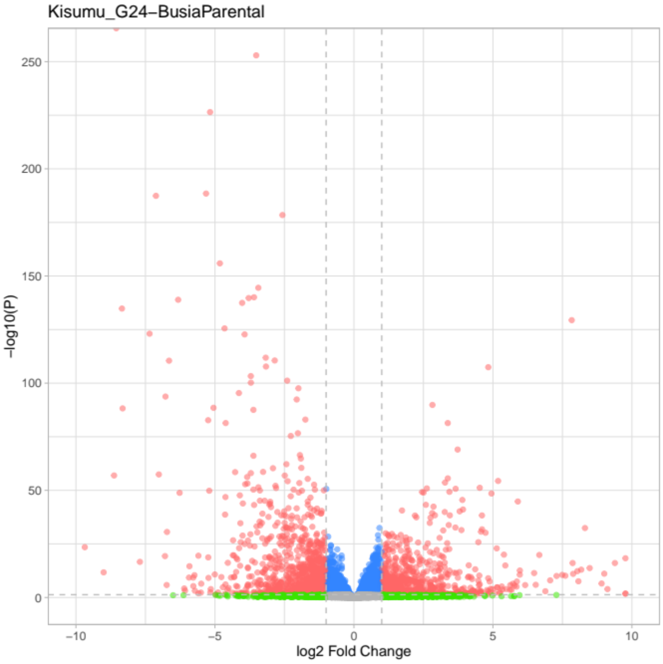

5) Heatmap of user-provided gene list

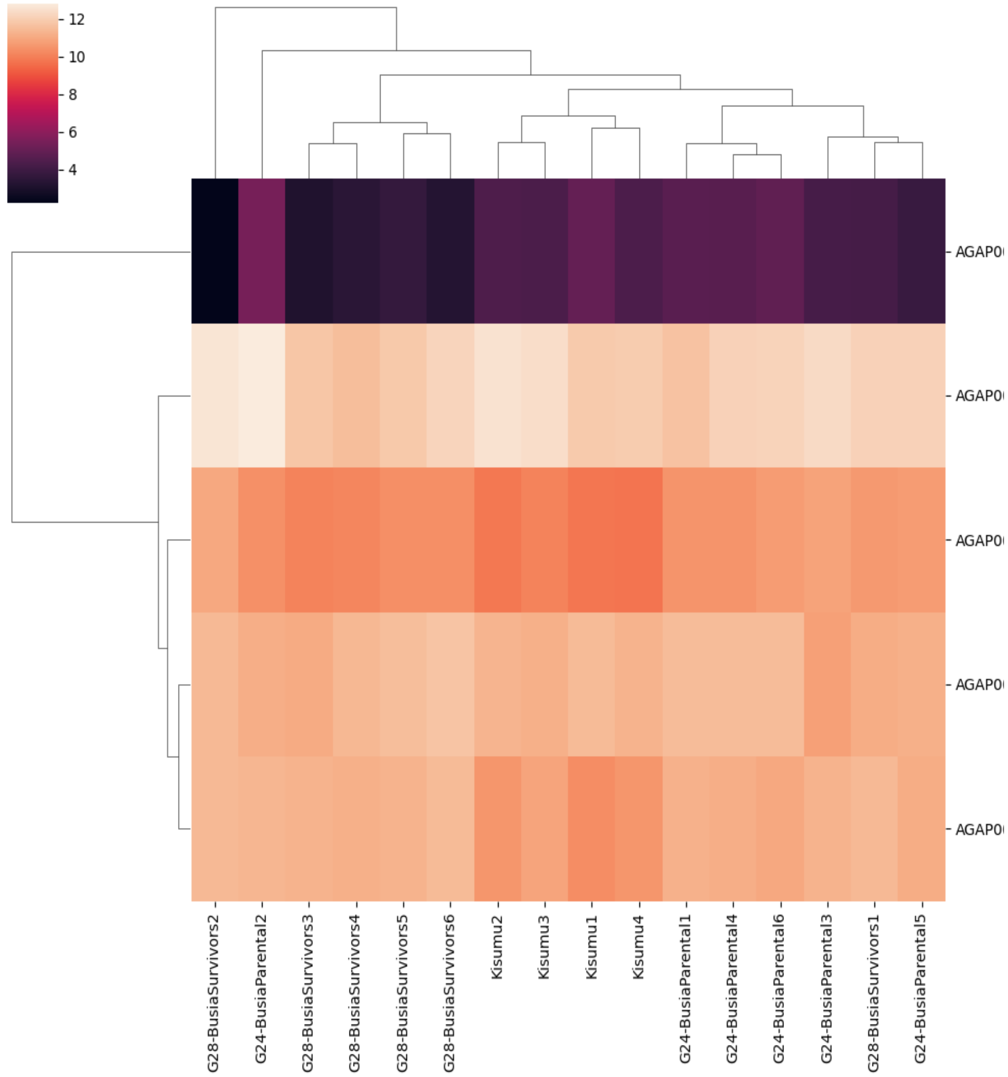

6) Venn diagrams

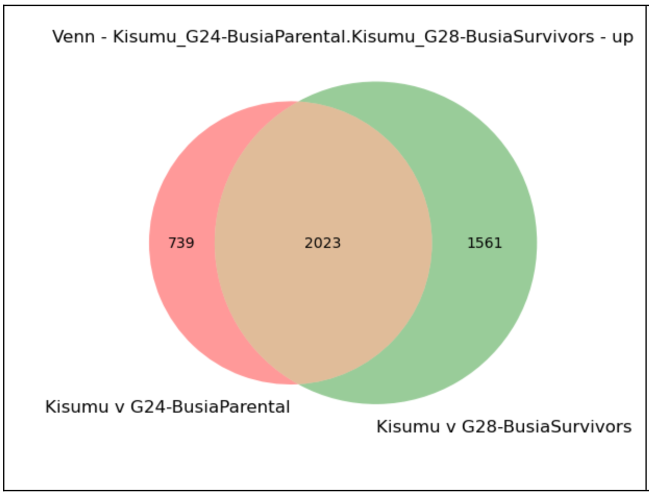

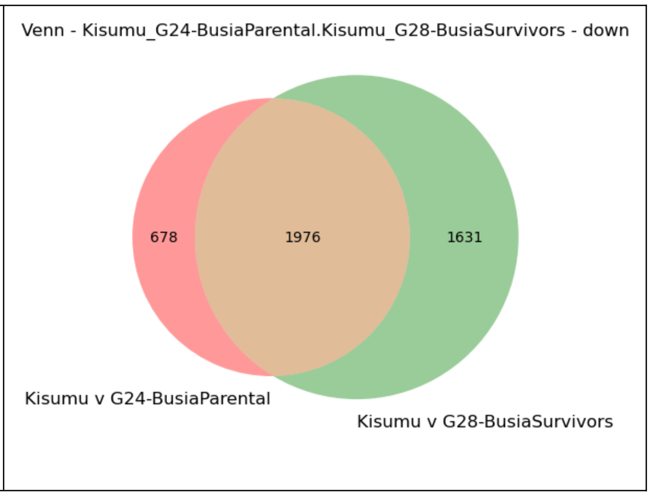

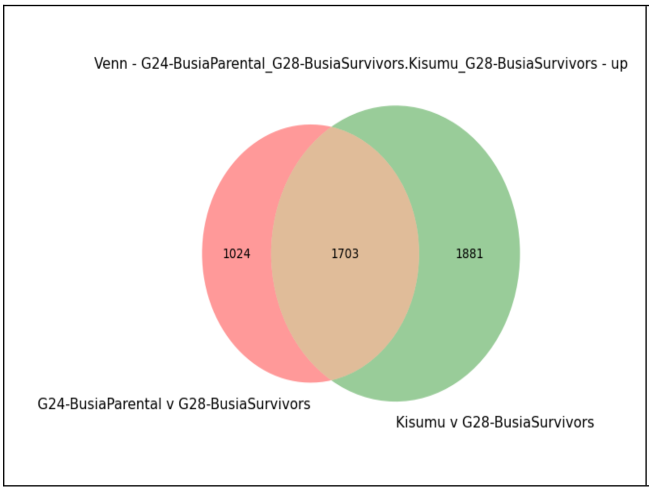

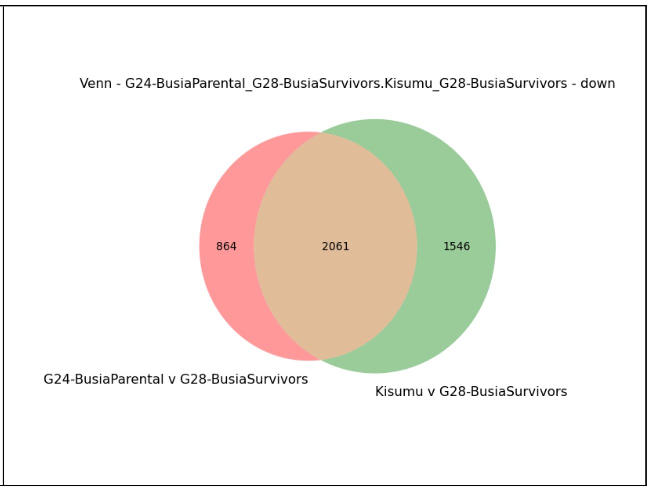

7) OLS

Regression of log2 number of SNPs per gene, total read counts per gene, and gene size in (bp).

```
 OLS Regression Results
==============================================================================
Dep. Variable:                  nSNPs   R-squared:                       0.431
Model:                            OLS   Adj. R-squared:                  0.430
Method:                 Least Squares   F-statistic:                     449.4
Date:                Fri, 21 Jan 2022   Prob (F-statistic):          5.48e-146
Time:                        13:11:34   Log-Likelihood:                -1734.4
No. Observations:                1189   AIC:                             3475.
Df Residuals:                    1186   BIC:                             3490.
Df Model:                           2
Covariance Type:            nonrobust
==============================================================================
                 coef    std err          t      P>|t|      [0.025      0.975]
------------------------------------------------------------------------------
const         -2.4515      0.257     -9.529      0.000      -2.956      -1.947
Readcounts     0.1462      0.011     13.698      0.000       0.125       0.167
GeneSize       0.4661      0.017     26.811      0.000       0.432       0.500
==============================================================================
Omnibus:                       87.964   Durbin-Watson:                   1.592
Prob(Omnibus):                  0.000   Jarque-Bera (JB):              106.486
Skew:                          -0.697   Prob(JB):                     7.53e-24
Kurtosis:                       3.457   Cond. No.                         160.
==============================================================================
```

Notes:
[1] Standard Errors assume that the covariance matrix of the errors is correctly specified.

8) PCA

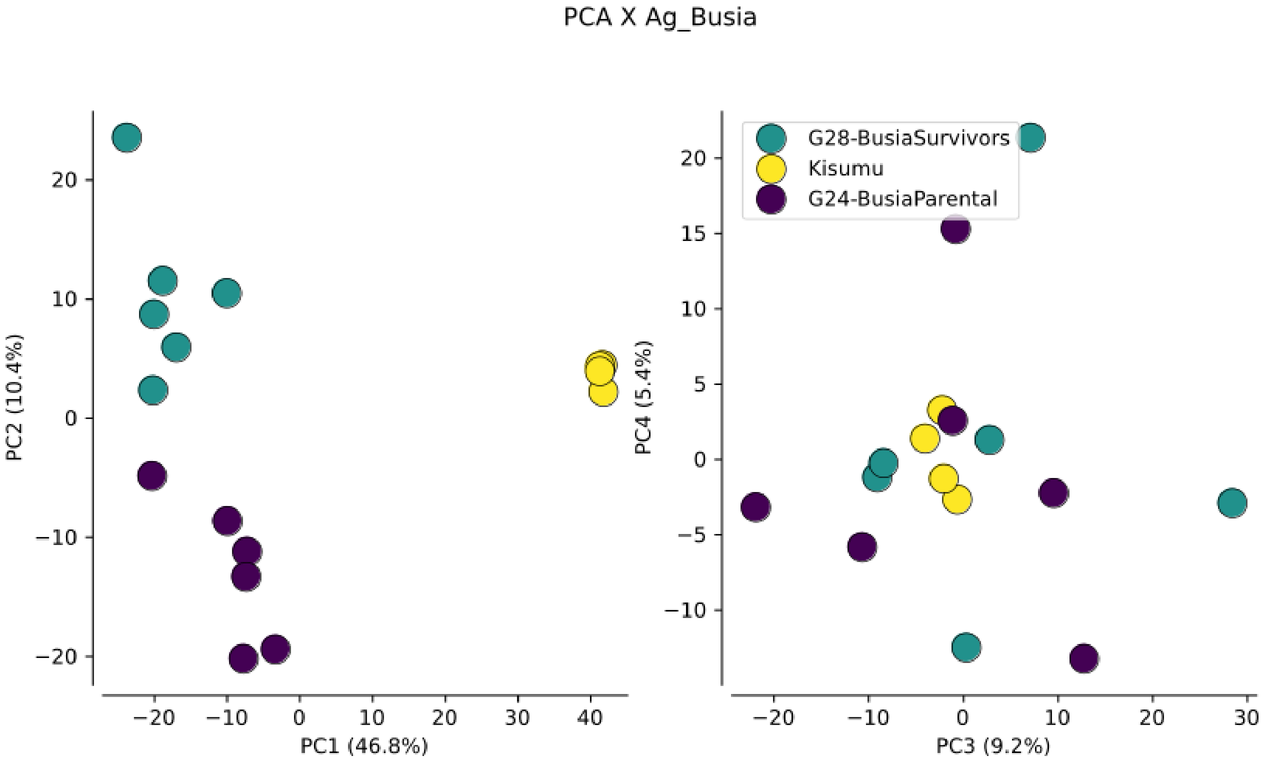
PCA X Ag_Busia
PC1 (46.8%)
PC2 (10.4%)
PC3 (9.2%)
PC4 (5.4%)
G28-BusiaSurvivors
Kisumu
G24-BusiaParental

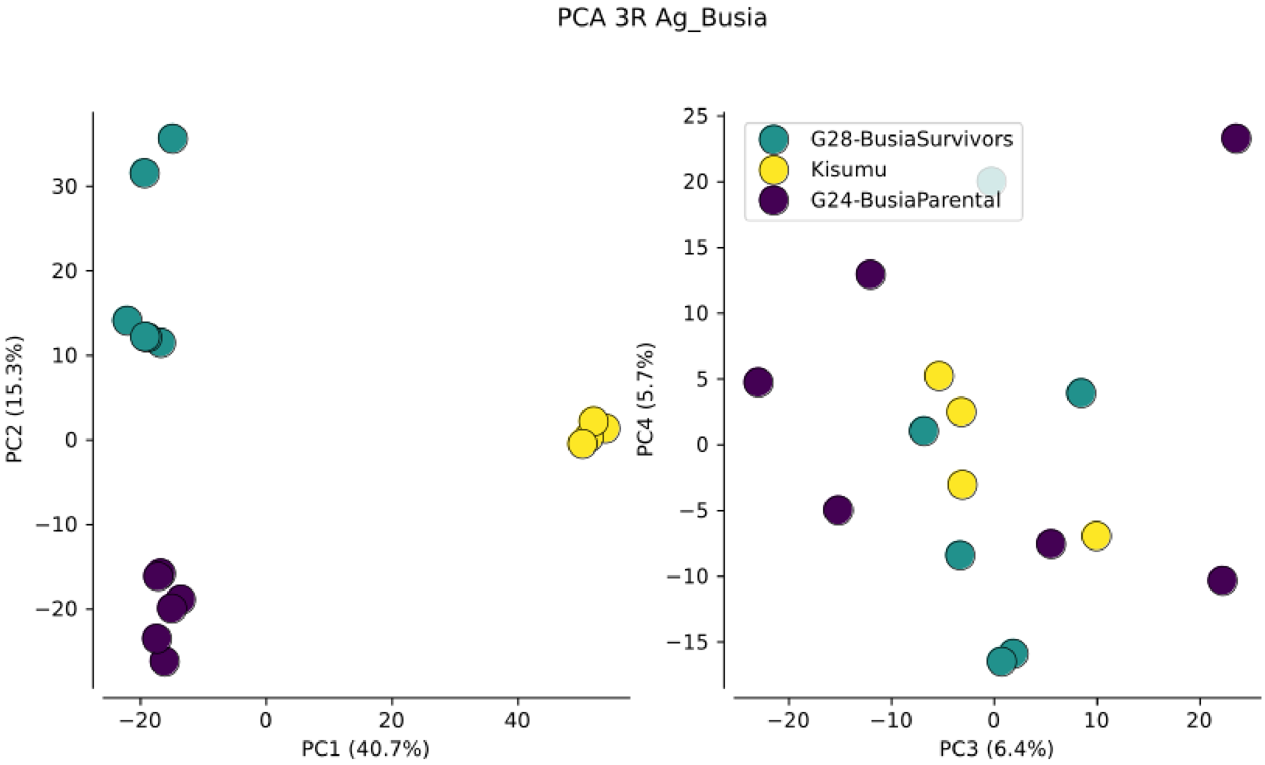
PCA 3R Ag_Busia
PC1 (40.7%)
PC2 (15.3%)
PC3 (6.4%)
PC4 (5.7%)
G28-BusiaSurvivors
Kisumu
G24-BusiaParental

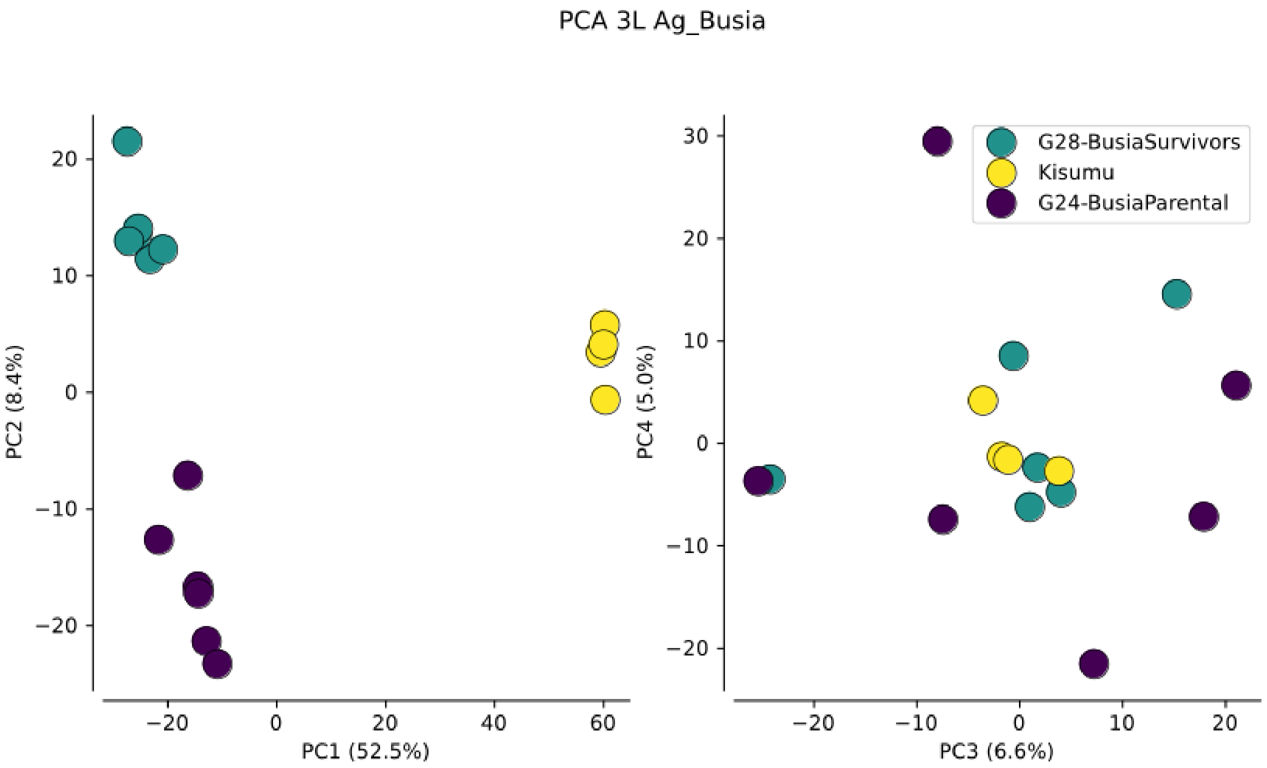
PCA 3L Ag_Busia
PC1 (52.5%)
PC2 (8.4%)
PC3 (6.6%)
PC4 (5.0%)
G28-BusiaSurvivors
Kisumu
G24-BusiaParental

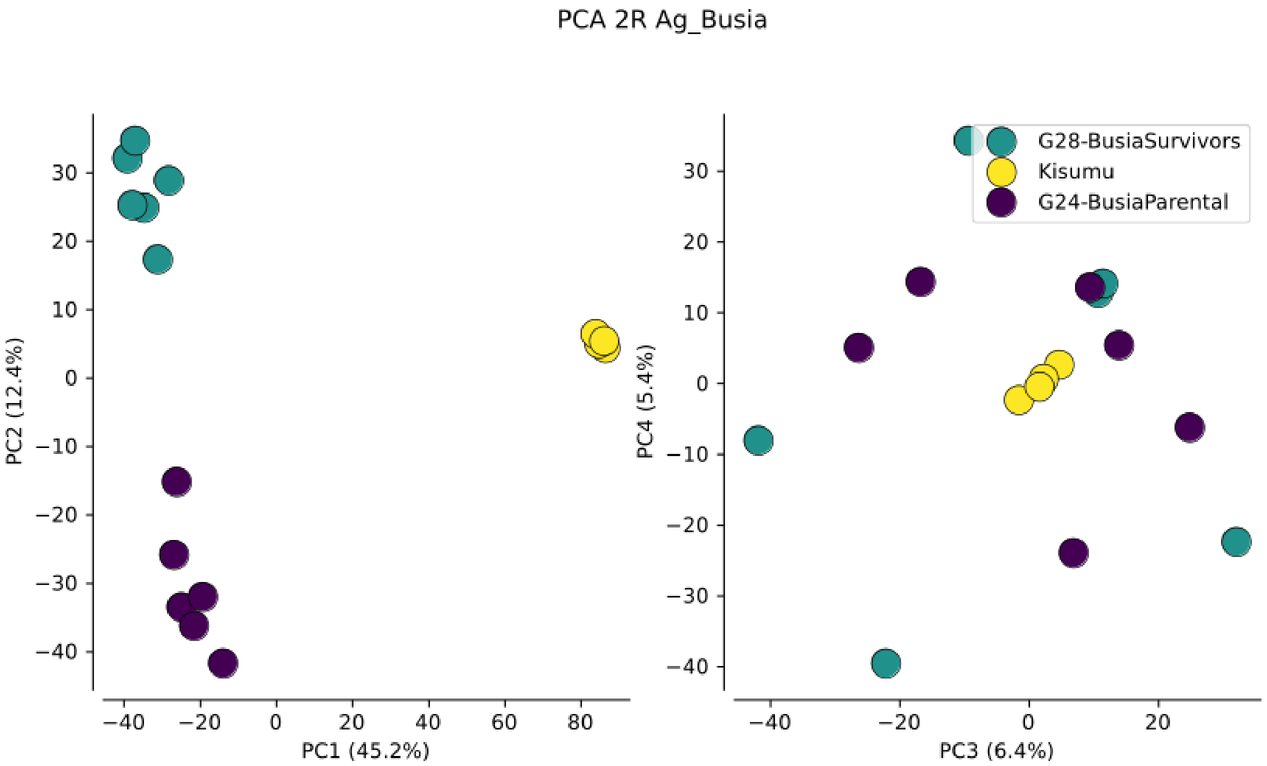
PCA 2R Ag_Busia
PC1 (45.2%)
PC2 (12.4%)
PC3 (6.4%)
PC4 (5.4%)
G28-BusiaSurvivors
Kisumu
G24-BusiaParental

PCA 2L Ag_Busia

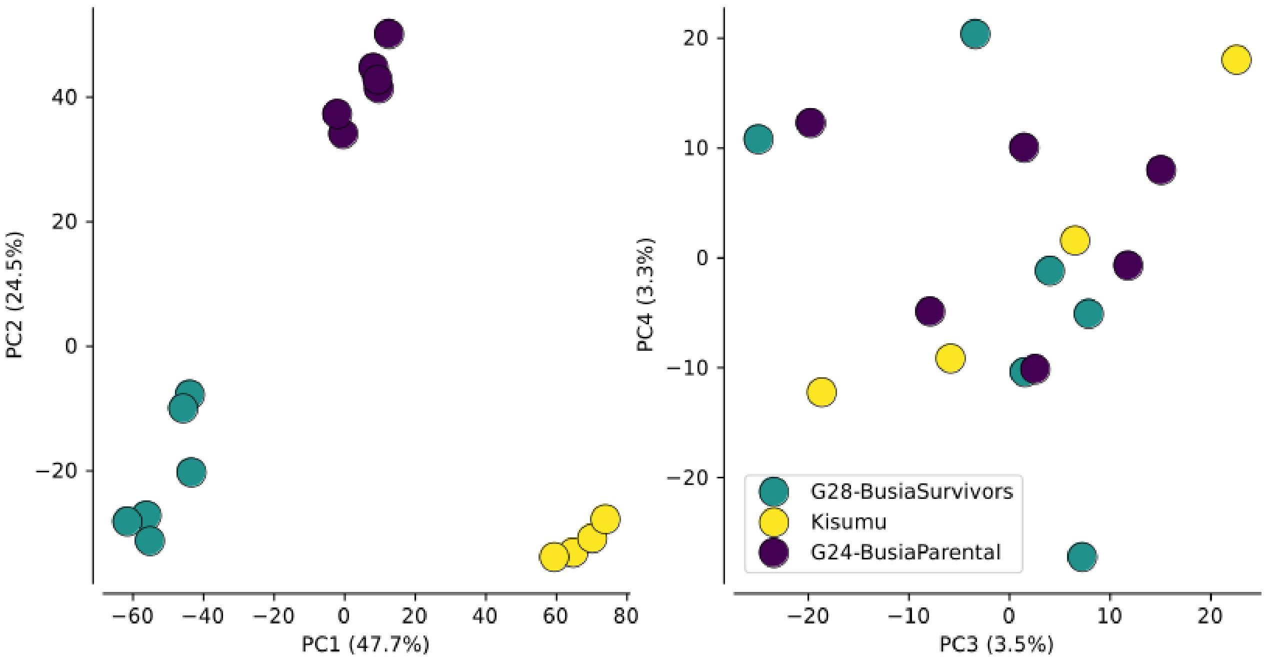

9) Genetic diversity

A) Wattersons Theta

Contig	G28 Busia Survivors	G24 Busia Parental	Kisumu
**2L**	0.00057	0.00068	0.00056
**2R**	0.00066	0.00083	0.00044
**3L**	0.00034	0.00059	0.00036
**3R**	0.00053	0.00067	0.00043
**X**	0.00068	0.00075	0.00025

B) Nucleotide Diversity

Contig	G28 Busia Survivors	G24 Busia Parental	Kisumu
**2L**	0.00053	0.00102	0.00084
**2R**	0.00097	0.00116	0.00065
**3L**	0.00038	0.00069	0.00048
**3R**	0.00075	0.00092	0.00064
**X**	0.00097	0.00123	0.00034

10) Hudson Fst Per chromosome (G24 Busia Parental v G28 Busia survivors)

Contig	Fst
**2L**	0.431
**2R**	0.109
**3R**	0.093
**3L**	0.11
**X**	0.106

11) Proportion of ancestry per chromosome

Strain	Contig	AIM fraction gambiae	AIM fraction coluzzii	n_aims
G28 Busia survivors	2L	0.981	0.000	53
G28 Busia survivors	2R	0.796	0.093	18
G28 Busia survivors	3L	0.871	0.062	15
G28 Busia survivors	3R	0.836	0.164	16
G28 Busia survivors	X	0.946	0.035	161
G24 Busia Parental	2L	0.981	0.000	53
G24 Busia Parental	2R	0.778	0.111	18
G24 Busia Parental	3L	0.887	0.047	15
G24 Busia Parental	3R	0.836	0.164	16
G24 Busia Parental	X	0.945	0.036	161
Kisumu	2L	0.105	0.875	51
Kisumu	2R	0.185	0.703	18
Kisumu	3L	0.057	0.877	15
Kisumu	3R	0.170	0.830	16
Kisumu	X	0.966	0.014	154

12 Karyotype frequencies calculated by compKaryo.

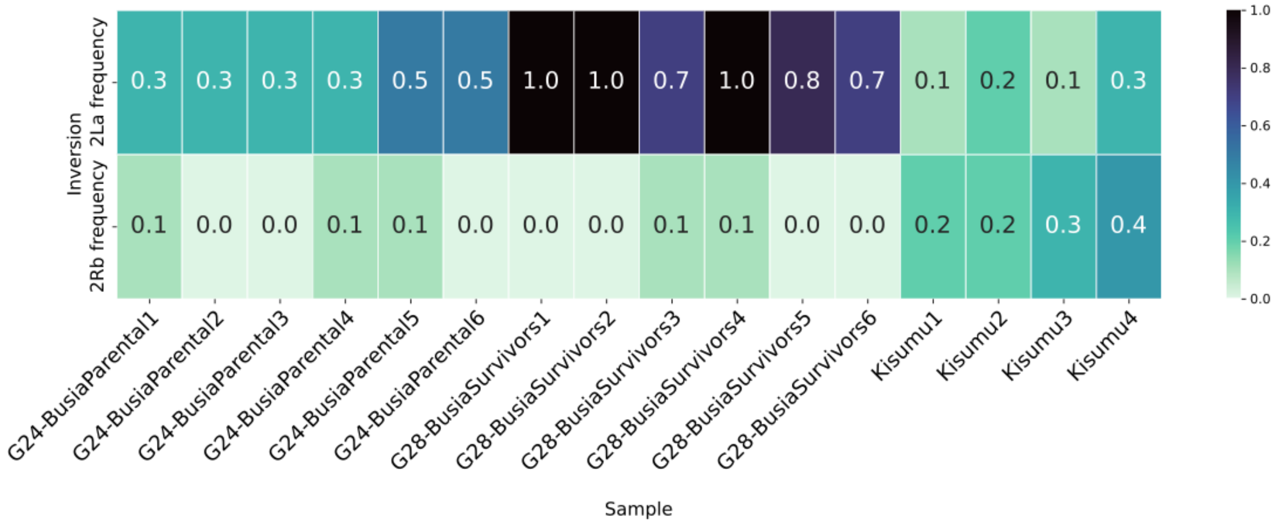

Supplementary Figure 12: Karyotype frequencies. The frequency of the 2La and 2Rb karyotypes in a) each biological replicate and b) averaged across experimental conditions
